# Designing Prepregnation and Fused Filament Fabrication Parameters for Recycled PP- and PA-Based Continuous Carbon Fiber Composites

**DOI:** 10.3390/ma17081788

**Published:** 2024-04-12

**Authors:** Marah Baddour, Ruth Garcia-Campà, Pablo Reyes, Dagmar R. D’hooge, Ludwig Cardon, Mariya Edeleva

**Affiliations:** 1 Centre for Polymer and Material Technologies, Department of Materials, Textiles and Chemical Engineering, Ghent University, Technologiepark 130, 9052 Zwijnaarde, Belgium; marah.baddour@ugent.be (M.B.); pablo.reyesisaacura@ugent.be (P.R.); ludwig.cardon@ugent.be (L.C.); 2 Applied Chemistry and Materials Department, Leitat Technological Center, C/Innovació 2, 08225 Terrassa, Spain; rgarcia@leitat.org; 3 Laboratory for Chemical Technology, Department of Materials, Textiles and Chemical Engineering, Ghent University, Technologiepark 125, 9052 Zwijnaarde, Belgium; dagmar.dhooge@ugent.be; 4 Centre for Textile Science and Engineering, Department of Materials, Textiles and Chemical Engineering, Ghent University, Technologiepark 70a, 9052 Zwijnaarde, Belgium

**Keywords:** 3D printing, polyamide (PA), polypropylene (PP), composite, continuous carbon fiber (cCF), enhanced flexural properties

## Abstract

Continuous carbon fiber (cCF)-based 3D-printed polymer composites are known for their excellent flexural properties; however, the optimization of the overall process is still desired, depending on the material types involved. Here, the improved manufacturing of cCF-based composites is reported, considering virgin polyamide (PA) and postindustrial waste polypropylene (PP), and the parameters affecting the material properties are evaluated. Firstly, the prepregnation technique was optimized to manufacture cCF polymer filaments with various fiber-to-polymer ratios. Secondly, the fused filament fabrication (FFF) technique was optimized. It was observed that the layer height needs to be sufficiently low for proper interlayer adhesion. The influence of the printing temperature is more complicated, with filaments characterized by a lower fiber-to-polymer ratio requiring a higher nozzle diameter and higher temperatures for efficient printing; and for lower diameters, the best flexural properties are observed for parts printed at lower temperatures, maintaining a high interspace distance. Plasma treatment of the cCF was also explored, as was annealing of the produced parts to enhance the flexural properties, the latter being specifically interesting for the PP-based composite due to a lower wetting caused by a higher viscosity, despite supportive interfacial interactions. Eventually, overall guidelines were formulated for the successful production of cCF-based composites.

## 1. Introduction

Additive manufacturing (AM) is a processing technique to fabricate parts with basic and complex shapes via layer-by-layer deposition of materials such as metals, polymers, and ceramics [1,2,3]. The advantages of AM, i.e., short design cycles, the possibility to manufacture complex structures, and high material utilization as compared to traditional manufacturing, have facilitated its wide application [4,5,6,7,8,9,10,11,12,13]. AM is specifically used for the processing of (thermoplastic) polymers and polymer-based composites, the latter being the focus of the present work, considering fused deposition modeling (FDM), which is also known as fused filament fabrication (FFF).

FDM/FFF is one of the leading AM processes based on extrusion and relies on the melting of a polymer filament in the extrusion nozzle and its subsequent deposition on a moving bed to create the final product layer by layer [5,6,7,14]. FFF has already established itself within the manufacturing industry [8,9]; nevertheless, the main limitation remains the relatively poor tensile properties of the printed products due to void formation [6,15,16]. Hence, it is worthwhile to, for instance, reinforce the printed parts, explaining why the production of 3D-printed polymer matrix composites with short or long continuous carbon fibers (cCFs) has grown [17,18]. It can be expected that such modification enables enhancement of the tensile properties and achievement of a high performance while maintaining the lightweight nature of the produced components in diverse industries.

cCF is often used as reinforcement for polymeric composites due to its low density, high strength, and high elastic modulus with applications for aerospace, windmills, the automotive industry, and sports appliances [15,16]. In general, cCF significantly enhances the tensile and flexural properties of the polymer composite parts [19]. As, e.g., shown by Isobe et al. [20], the utilization of cCF allowed for a seven-times increase in tensile strength and a five-times increase in elastic modulus. More recently recycled products could be made as well, broadening the market potential [19]. For instance, Alarifi et al. [21] showed that cCF-based composites from virgin and recycled polyethylene terephthalate glycol (PETG) have comparable thermal, mechanical, and rheological properties. The authors also showed that recycled PETG has better interfacial interactions with cCF, due to a lower viscosity. Consistently, Tian et al. [22] reported the production of recyclable cCF-poly(lactic acid) (PLA) composites with 100% cCF and 73% PLA recovery potential.

Another important characteristic of cCF, highlighted by Li et al. [23,24], is its superior fatigue and corrosion resistance when compared to traditional metal materials. This quality explains why cCF-based composites are widely used in various anchoring technologies.

**Figure 1:**
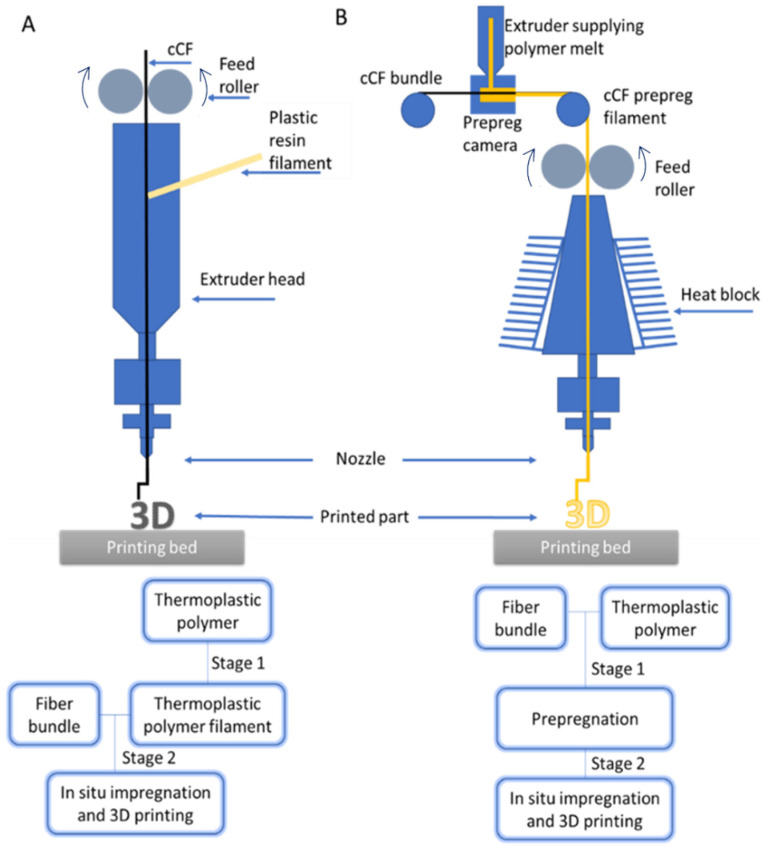
Two techniques were used to print continuous carbon fiber (cCF)-reinforced polymers. (**A**) Set-ups. (**B**) General descriptions.

As shown in Figure 1, two techniques are typically used to exploit the FFF principle to produce cCF-reinforced composites [22,25], i.e., a technique based on in situ (in-printhead) melt impregnation [26] and a technique based on pre-impregnation followed by 3D printing [27]. During in situ melt impregnation (Figure 1A), the cCF is dragged through the die of an extruder, which melts the matrix material typically in filament form, toward simultaneous composite production and printing. For the pre-impregnation technique, the cCF polymer filament is first manufactured in a separate device and then the filament is used for the actual 3D printing [27,28]. Note that both techniques are two-stage, as highlighted in Figure 1B. However, the pre-impregnation-based process gives more control over the quality of the produced filament, as the so-called prepreg parameters can be varied, ensuring a good wetting of cCF with the polymer [29,30], playing with different polymer-to-cCF ratios [31].

One of the ways to enhance interfacial interactions during pre-impregnation is cCF plasma treatment [32]. For instance, Yuan et al. [33,34] showed that plasma treatment increased the surface roughness of the carbon fibers and changed the functional groups on the surface. Cho et al. [35] additionally showed that plasma treatment significantly increased the storage modulus and impact strength of CF–polycarbonate composites. Furthermore, Ma et al. [36] studied the effect of oxygen plasma treatment on the interface of CF–epoxy composites. The authors highlighted that the treatment enhanced the surface roughness and increased the surface free energy by 45% after 3 min of plasma treatment. Another parameter that can influence the wetting of cCF by the matrix is the viscosity of the polymer. If the viscosity is too high, the wetting is rather ineffective, even in the case of favorable interfacial interactions from a functional group point of view [37,38].

It should be admitted that it remains a challenge to develop an impregnation device that allows the production of prepreg filaments at high speeds and low cost [39]. The prepregnation system should allow sufficient contact of the cCF with the polymer melt, knowing that the nozzle diameter of the prepregnation chamber influences the ideal polymer-to-cCF ratio. Installing a good winding system is also important, as the diameter of the produced prepreg filament is small, making it very brittle.

To engineer a proper contact of the cCF with the polymer melt, three main techniques exist, namely, the pultrusion, passive pin, and active pin techniques [40]. The pultrusion technique is applied in a liquid matrix medium which can be a monomer precursor, a solution, or a polymer melt, whereas the passive pin technique uses the generated pressure between the spreader pin and the fiber bundle in the melt chamber as a driving force for the proper cCF impregnation. The active pin technique involves the injection of a liquid/molten polymer between a bundle of fibers and a cylindrical friction surface to ensure that the entire volume of the matrix material is impregnated with cCF.

It should be further realized that the prepregnation is only one step in the right-hand part of Figure 1, and one should also put emphasis on the optimization of the FFF process as such, i.e., the tuning of the layer height (*h*), the nozzle temperature (*T_n_*), and the printing pattern, to minimize voids during printing. In this framework, Kuznetsov et al. [41] investigated the effect of *h* on the flexural strength ($\sigma_{flex}$) of PLA printed parts, showing that a smaller *h* provides a higher $\sigma_{flex}$ and implicitly lesser voids. Similarly, Wang et al. [42] highlighted the better tensile, flexural, and impact strength of 3D-printed polyetheretherketone (PEEK), CF/PEEK, and glass fiber/PEEK parts. Hu et al. [27] showed that *h* significantly influences the final strength and modulus of PLA-based parts, while the printing temperature and speed have only a minor effect. Li et al. [43], in turn, demonstrated that when the printing temperature increased from 180 °C to 230 °C, $\sigma_{flex}$ and the flexural modulus ($E_{flex}$) of PLA printed parts increased by 46% and 32%, but as soon as *h* increased from 0.35 mm to 0.55 mm, the flexural properties of the specimens decreased dramatically. In agreement with the above findings, the model of Garzon-Hernandes et al. [44] predicts an increase in tensile strength in acrylonitrile–butadiene–styrene (ABS) polymer parts with a lower *h*.

**Figure 2:**
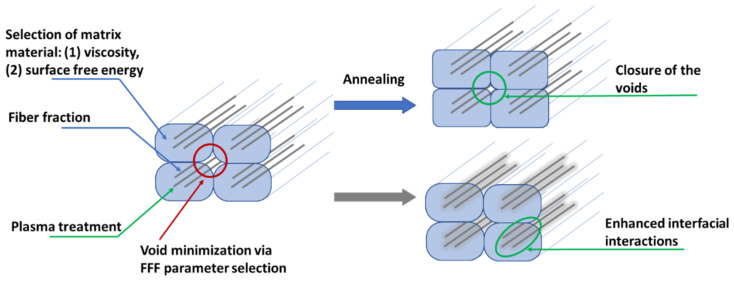
Methods for the enhancement of the material properties of cCF-based printed materials during different steps in Figure 1 or via post-treatment.

Complementary material design can also be performed after the FFF or 3D printing step, as the 3D-printed parts can be subjected to a post-treatment, e.g., annealing, to induce closure of the voids, adhesion of the layers, and recrystallization of semi-crystalline polymers and residual stress release of amorphous ones [45,46]. By annealing above the glass transition temperature (*T_g_*) and below the melt temperature (*T_m_*) for amorphous polymers, or below the recrystallization temperature (*T_cc_*) for semi-crystalline thermoplastics, the void content can be significantly decreased [47]. Notably, Bhandari et al. [48] explained that the main mechanism of tensile property increase by annealing is caused by the better incorporation of polymer molecules between layers, providing adhesion, rather than by increasing the crystallinity. Typically, annealing has only a positive effect on the mechanical properties if a slow cooling is applied [48]. Hence, to adjust cCF-reinforced polymeric parameters, several design steps can be conducted, as summarized in Figure 2.

In this contribution, cCF-based composite filaments were manufactured with a recycled polypropylene (PP) or a virgin polyamide (PA) matrix. The prepregnation filament fabrication technique was employed to produce the parts, employing an in-house machine for the prepregnation process with an enhanced active pin system. The processing parameters for prepregnation of the filament were systematically varied (e.g., nozzle diameter (*D*)), as were the conditions for FFF (e.g., layer height (*h*), interpath distance (*s*), and nozzle temperature (*T_n_*)), to gain an understanding of their influence on the flexural properties of the resulting parts. To enhance the quality of the filaments, the influence of cCF plasma treatment was also studied, along with an exploration of the impact of annealing on the morphology of the printed parts and their flexural properties. In other words, all optimization methods shown in Figure 2 were addressed, making the current work detailed and complementary to the state of the art.

Based on the overall findings, general guidelines for 3D printing-based manufacturing of cCF-reinforced composite parts for both virgin and recycled polymers were formulated.

## 2. Materials and Methods

### 2.1. Materials

Sabic PP 108MF10 postindustrial clean waste polypropylene (PP) from car parts supplied by Maier s.coop group (Bizkaia, Spain), and Rilsamid^®^ AMNO TLD–PA12 from Arkema (Colombes, France) were used as polymer matrix materials. Torayca^®^ T300B–3000 yarn from Toray Composite Materials America (Inc, Tacoma, WA, USA), containing an average of 3000 individual fibers, was used as the cCF. Table S1 in the Supplementary Materials shows an overview of the most relevant material properties for the matrix materials and cCF.

### 2.2. Manufacturing cCF Prepreg Filaments

**Figure 3:**
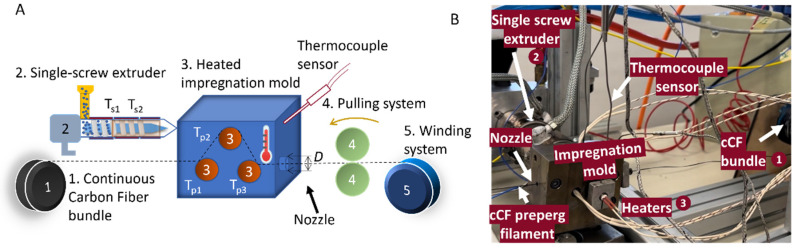
(**A**) Concept of the pre-impregnation process, starting from the principles introduced in Figure 1. (**B**) Actual implementation. 1: Continuous carbon fiber (cCF) bundle, 2: single screw extruder with two heating zones (*T_s_*_1_ and *T_s_*_2_), 3: heated impregnation mold with 3 impregnation pins (*T_p_*_1_, *T_p_*_2_, and *T_p_*_3_), 4: pulling system which controls the pultrusion speed (*V_pultrusion_*), 5: winding system.

cCF prepreg film manufacturing was conducted on an in-house machine, with the main components displayed in Figure 3 [49]. The machine consists of a single screw micro-extruder (element 2 in Figure 3) with two temperature zones (*T_s_*_1_ and *T_s_*_2_) [50] which delivers molten polymer to a heated mold (element 3 in Figure 3). To further optimize the prepreg process, three active temperature-controlled pins were introduced (temperatures *T_p_*_1_, *T_p_*_2_, and *T_p_*_3_), which are equipped with a slit to inject the liquid polymer between the cylindrical contact surface and the fiber bundle to avert the dry contact between the fiber and the spreader pin, as shown in Figure S1 in the Supplementary Materials. The pulling system (elements 1 and 4 in Figure 3) pulls the cCF through the polymer melt in the impregnation mold (element 3 in Figure 3). The produced filament is then mounted on a spool by a winding system (element 5 in Figure 3) in view of subsequent FFF. The system is controlled by a Programmable Logic Controller (PLC) through TwinCAT3 software (Version 3.1, Build 4024.56), which gives real-time data and feedback through sensors.

For the filament production, the extruder screw temperatures were optimized (*T_s_*_1_ and *T_s_*_2_, element 2 in Figure 3), as were the rotational speed of the extruder (*N*, element 2 in Figure 3), the temperature of the impregnation chamber (*T_C_*, element 3 in Figure 3), the temperature of the impregnation pins (*T_p_*_1_, *T_p_*_2_, and *T_p_*_3_; element 3 in Figure 3), and the pultrusion speed (*V_pultrusion_*, elements 1 and 4 in Figure 3).

The heated impregnation chamber is also equipped with nozzles with variable diameters, which control the diameter of the prepreg filament and consequently the polymer-to-fiber fraction. Four nozzle diameters were used (*D*, element 3 in Figure 3) for PA- and PP-based prepreg filaments, namely, 1.1 mm, 0.9 mm, 0.7 mm, and 0.6 mm. The PA fiber volume fractions ($V_{f}$) were, e.g., 13.5%, 18.7%, 30%, and 37.6%, respectively. These fiber volume fractions were measured experimentally by thermal degradation of the polymer matrix and weighing the remaining fibers using sample sizes ranging from 500 mg to 5 g of the initial composite material and performing the degradation below 500 °C in a vacuum oven for 2 h. The following equations were used to calculate the filler volume fraction:

(1) $$M_{f}=\frac{m_{f}}{m_{c}}$$

(2) $$V_{f}=\frac{\rho_{m}\;M_{f}}{\rho_{f}+M_{f}\left(\rho_{m}-\rho_{f}\right)}$$

where *m_c_* is the composite mass [kg], *m_f_* is the filler mass [kg], *M_f_* is the filler mass fraction [-], $\rho_{f}$ is the filler density [kg m^−3^], $\rho_{m}$ is the matrix density [kg m^−3^], and *V_f_* is the filler volume fraction [-] [49].

Table 1 lists the specifications of the 17 filaments produced with the optimized prepreg parameters. The variables were determined through trial and error by testing various parameters to identify the optimal ones.

### 2.3. Fused Filament Fabrication of cCF Prepreg Filaments

**Figure 4:**
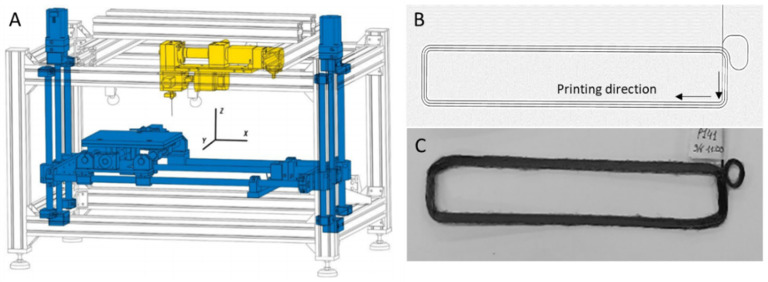
(**A**) Sketch of in-house FFF printer. The bed and driving system are in blue, and the extruder is in yellow. (**B**) Top-view sketch of the toolpath of the printed specimens. (**C**) Actual printed part (printing condition P141 in the Supplementary Materials).

For FFF, an in-house developed machine was used (Figure 4A) consisting of an aluminum printing bed coated with polyetherimide moving in the X, Y, and Z directions, with a printing volume of 250 × 200 × 145 mm^3^. The printing chamber is heated by two infrared radiators mounted below the nozzle. The machine is insulated by using an insulator wall of 5 cm thickness from Recticel (Wetteren, Belgium) to minimize warping and improve layer adhesion. During FFF, the layer height (*h*), interpath distance (*s*), and nozzle temperature (*T_n_*) were controlled. The nozzle temperature was chosen between 200 and 235 °C. The bed temperature was set at 110 °C for cCF-PA and ranged from 60 to 110 °C for cCF-PP (see Table S8 in the Supplementary Materials).

It should be further noted that the printing of cCF prepreg filaments could not be performed with a zig-zag pattern to obtain a complex shape (e.g., a dog-bone shape for the tensile test) because of the high stiffness of cCF. Hence, rectangular specimens were printed, as shown in Figure 4B. The individual tracks of the test specimens were deposited in the same direction due to the spiraling pattern. Upon adding more than one layer, a small loop was made to start a new layer using the same X and Y pattern.

To optimize *h* and minimize voids, the long straight paths were printed at 120 mm/min, and the curves and short sides were printed between 30 and 60 mm/min. The actual view of the printed specimen is shown in Figure 4C. All printing conditions are summarized in Table S8 in the Supplementary Materials, while Table 2 lists examples of key printing parameters that were used for the production of the parts (notation with P), starting from filaments listed in Table 1 (notation with F).

### 2.4. Plasma Treatment

Certain cCF materials were treated in continuous mode using an Atmospheric Pressure Glow Discharge (APGD) device, model PLATEX 600–LAB VERSION, obtained from Grinp, S.r.l. (Settimo Torinese, Italy). The two-planar electrode equipment operated with a frequency range from 20 to 45 kHz to partially ionize gases and/or vapors of precursors. Two types of plasma treatment were used, namely, oxidation with He/O_2_ and etching with He/Ar gases. The flow was kept at 2.5/1.5 L/min in both cases, and the power was set to 700 W. The speed of the cCF pultrusion was 1 m/min.

After plasma treatment of cCF, the pre-impregnation process was run with PP as a matrix material to produce the prepreg filaments F002 and F003 in Table 1 with oxygen–helium plasma- and argon–helium plasma-treated cCF, respectively. Conditions P093 and P095 were used for FFF, incorporating oxygen–helium plasma and argon–helium plasma treatments for cCF, respectively, as shown in Table 3, which also includes the reference untreated case.

### 2.5. Annealing

The printed specimens for both polymer types were annealed in a vacuum oven for 3 h (excluding heating time). All annealed selected specimens were compared to the unannealed specimens with the same printing parameters, as shown in Table 4.

### 2.6. Rheological, Morphological, and Thermal Property Characterization

Rheological measurements for the PP matrix material were performed for the compression-molded disk-shaped specimens with a 25 mm diameter and a 1 mm thickness manufactured in a hot press (Fontijne Holland, Vlaardingen, The Netherlands) at 200 and 230 °C. The frequency sweep tests were performed in an MCR 702 rheometer (Anton-Paar, Graz, Austria), using the parallel plate configuration with a 25 mm diameter and a 1 mm gap. The complex viscosity (η*) was monitored as a function of the angular frequency (from 600 to 0.1 rad/s), with a strain amplitude of 0.1%, under a nitrogen atmosphere. The strain amplitude was defined employing amplitude sweep tests, and all the materials were assumed to be tested in the linear viscoelastic regime. All materials were dried at 60 °C in a vacuum dryer overnight prior to molding and rheological testing. The rheological data for PA12 were obtained from the datasheet.

Optical microscopy for the printed parts was performed on a VHX-7000 Keyence OM (Keyence International NV/SA, Mechelen, Belgium) and applying its accompanying software. The obtained images from the OM were subsequently subjected to processing and analysis through the ImageJ software (Version 1.51), to evaluate the voids volume. Scanning electron microscopy (SEM) images were obtained on a Phenom Pro electron microscope (Benelux Scientific, Ede, The Netherlands). The samples were coated with a 10 nm gold layer, and images were taken at 5 kV.

The thermal properties of the polymer matrices and the prepreg filaments were studied via differential scanning calorimetry (DSC) with a DSC 214 Polyma device (NETZSCH-Gerätebau GmbH, Selb, Germany). All the materials were heated up from 25 to 250 °C with a 10 °C/min ramp considering two heating–cooling cycles. Thermographic analysis (TGA) was performed on a Netsch STA 449 F3 (NETZSCH-Gerätebau GmbH, Selb, Germany). Tests were performed under a nitrogen (N_2_) atmosphere, with a flow rate of 50 mL·s^−1^ and a heating rate of 10 °C·min^−1^, according to the standard ISO 11358-1 [51]. All samples had an initial mass of ca. 10 mg.

### 2.7. Flexural Property Measurements

The test samples were cut from the printed specimens (Figure 4B,C) according to ISO 14125 [52] (Table S2). All flexural tests were performed for samples that were conditioned for 48 h in the lab before conducting the “climatized samples” test, using a three-point flexural testing set-up and a 2 kN load cell on an Instron 4464 machine at a rate of 1 mm/min. The flexural modulus ($E_{flex}$) was calculated to be between 0.15% and 0.20% strain. The values mentioned for $\sigma_{flex}$ are the maximal stress values of the flexural curves, which are typically reported for continuous fiber composites [49].

## 3. Results and Discussion

To gain an understanding of the influence of the composition on the manufacturing process, 17 filament formulations were prepared (see Table 1) with varying matrix materials (PP or PA12) and fiber volume fractions ($V_{f}$). These formulations were used for printing according to 215 conditions (see Table S8 in the Supplementary Materials) to identify relations between printing and overall process parameters and morphology, including plasma treatment and annealing. In what follows, the main results are discussed, focusing first on the wetting potential, both theoretically and by morphological analysis, and then on the void minimization by tuning the process parameters, and finally on the tuning of flexural properties.

### 3.1. Wetting Degree after Impregnation

The interfacial interactions can be theoretically assessed based on the surface free energies of the matrix and filler, $\gamma_{A}$ and $\gamma_{B}$, respectively. In general, the surface energy of the filler should be greater than that of the matrix for proper wetting to take place. For the chosen cCF and the selected matrix polymers, data in the literature suggest that this is the case, with the PA12 *γ_PA_* being equal to 40 mN/m [53] and the PP *γ_PP_* being equal to 30.1 mN/m [54], both lower than the *γ_CF_* of 53 mN/m [55]. Thus, from a theoretical standpoint, both polymers should demonstrate good adhesion to cCF.

**Figure 5:**
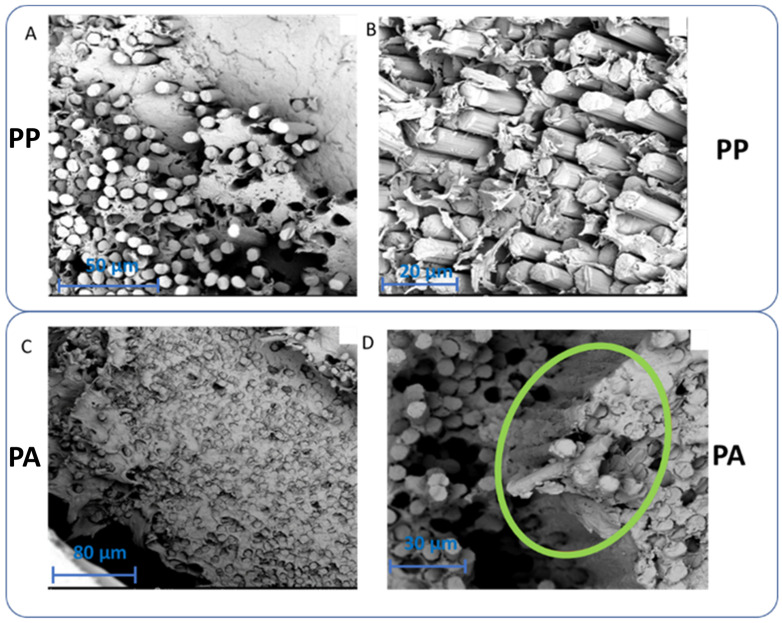
SEM images of the compression fracture surface of cCF/polypropylene (PP) (**A**,**B**) and cCF/polyamide (PA) (**C**,**D**) printed parts. (**A**) Filament F001, printing condition P002. (**B**) Higher magnification of image (**A**). (**C**) Filament F013, printing condition P126. (**D**) Higher magnification of image (**C**). The green oval shows the (almost) complete wetting of the fiber with the polymer matrix.

However, the SEM analysis in Figure 5A,B shows deviation from this theoretical insight, as the compression fracture of CF-PP displays a clear separation of the individual fibers and matrix, indicative of poor wetting. In more detail, a delamination zone inside the fiber bundles was observed, which was due to inefficient wetting causing the fibers to break during printing (Figure S2b in the Supplementary Materials). On the contrary, PA12 followed the theoretical insights and showed much better adhesion to cCF (Figure 5C,D).

A good dispersion of cCF in the matrix can be observed here and a very flat fracture surface, where the fiber and matrix broke as a whole (Figure 5D), which shows that individual fibers are (close to) fully coated with PA12, which was not observed for PP. Additional SEM images (Figure S3 in the Supplementary Materials) for the PA12-based composite further support the good wetting of the fibers with the matrix material. Evidently, on the microscopic level, PA12-based composites possess superior properties due to the proper wetting of the fibers by the matrix.

Another parameter to consider for the optimization of the wetting is the viscosity of the polymer melt, with a lower viscosity facilitating the wetting. Figure S6 in the Supplementary Materials shows that the viscosity of PP is significantly higher than that of PA, disfavoring the wetting of the cCF with PP, as was observed via SEM. Note that in the theoretical calculations, such viscosity variation is not included, at least partially explaining the differences with the SEM images. For further investigation, the estimated shear rate (γ˙w) for PP during the printing process was calculated at three different temperatures, 215 °C, 225 °C, and 235 °C, employing the following equations [56]:

(3) $$\gamma ˙a=\frac{4Q}{\pi R^{3}}\;\left[s^{-1}\right]$$

(4) $$\gamma ˙w=\frac{\gamma ˙a}{4}\;(3+\frac{1}{n})\;[s^{-1}]$$

where *Q* represents the volumetric flow rate, which is derived from the melt density (*ρ*) and the weight per unit time discharged from the printer at the used extrusion speed. The melt density (*ρ*) was obtained from the melt flow index test, the nozzle diameter was 2*R* = 2 mm, and *n* was the power law index determined from rheological data, taking on a value of *n* = 0.2 for PP. Table S3 in the Supplementary Materials shows low values for the estimated shear rate (γ˙w). Therefore, the lack of wetting in the cCF-PP specimens was due to high viscosity. Additionally, the voids analysis showed a slight reduction in void content for samples at higher temperatures (235 °C) compared to samples printed at 225 °C (see Table S4 in the Supplementary Materials). This decrease is attributed to a lower viscosity, facilitating improved wetting between the matrix and the filler.

### 3.2. Optimization of FFF Parameters to Decrease Voids

**Figure 6:**
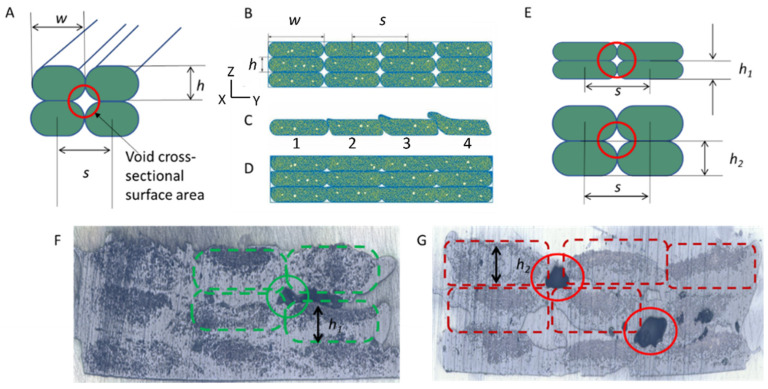
(**A**) Theoretical cross-section of the 3D-printed incompressible printed specimen. *w*—the width of an individual strand, *s*—the distance between two tracks, *h*—the layer height; the void cross-sectional surface area (highlighted with a red circle) is star-shaped. (**B**–**D**) Influence of the void filling factor on the cross-section of the 3D-printed parts: (**B**) *s* = *w*, ξ = 0; (**C**) *s << w*, ξ = 1; (**D**) *s < w*, ξ = 0.5. (**E**) Dependence of the void cross-sectional surface area on the layer’s height for smaller (**upper image**) and larger (**lower image**) *h* values. Optical images of (**F**) cross-section of CF-PA12-based part, with *s* = 1.37 mm and *h* = 0.29 mm (printing condition P126), and of (**G**) cross-section of CF-PA12 specimen, with *s* = 1.06 mm and *h* = 0.22 mm (printing condition P154). Colors used to facilitate identification of printing quality, with green indicating better quality.

Due to the layer-by-layer nature of FFF with imperfect interlayer adhesion, the 3D-printed part may contain voids, which can affect the material properties, justifying the control of printing parameters to reduce the void content and sizes. The filaments were first theoretically considered to be incompressible solids with an elliptical cross-section so that the void cross-section would have a four-arm star shape (Figure 6A). These macro-void volume fractions (Vmac) exist between the individual deposited tracks and are determined using Equation (5). By reducing the ratio of the layer thickness (*h*) to the individual strand width (*w*), Vmac can be reduced. Additionally, by adjusting *s*, which is the distance between two tracks, the shape and the content of these voids can change. In particular, Equation (6) can be used for the connection of *s* and *w*:

(5) $$Vmac=\frac{h}{w}(1-\frac{\pi }{4})$$

(6) $$s=w-\xi \cdot h\cdot (1-\frac{\pi }{4})$$

where ξ is the void-filling factor, which ranges from 0 to 1. The complete derivation of Equation (6) is included in the Supplementary Materials.

Figure 6B shows a theoretical situation in which filament strands are deposited next to each other, with *w* = *s* and ξ = 0, which is the case for an incompressible filament with star-shaped voids. If ξ = 1 (Figure 6C), the nearby tracks overlap too much, decreasing the quality of the printed part. If ξ = 0.5 (Figure 6D), a good balance between void filling and printability can be achieved [49]. Additional discussion of the influence of ξ on the void content is provided in the Supplementary Materials. Notably, *h* affects the void size as well, with Figure 6E making it theoretically evident that the voids between layers with different thicknesses diminish if the layer thickness becomes smaller. Figure S9 in the Supplementary Materials shows a comparison between the calculated and experimental impact of *h* on the void content. In general, *h* is determined by the filament diameter (*D*), by the distance between the printing head and the bed, and by the deformation of the filament during printing. Furthermore, with a larger *s*, the filament has more room to deform during melting, which results in smaller voids as well.

In view of the theory mentioned above, four nozzle diameters were selected (*D* = 1.1 mm, *D* = 0.9 mm, *D* = 0.7 mm, and *D* = 0.6 mm) to study the influence of *h*. Optical microscopy was used to study the effect of the printing conditions on the quality of the part and voids content. Figure 6F,G, for example, show optical microscopy images of a three-layered CF-PA12 specimen with *s* = 1.37 mm and *s* = 1.06 (specimen F013, printing conditions P126 and P154; Table 2). Indeed, a denser composite with a lower void content was observed when *h* was smaller (Figure 6F). Upon exploring the range of printing conditions, *s* could be gradually brought to 1.2 mm, at which point both cCF-PP and cCF-PA12 experienced complete breakage of the filament during printing. A complete overview of the results for all printing conditions is given in Table S8 in the Supplementary Materials. More optimal parameters always include a larger *s* and a smaller *h*.

### 3.3. Optimal FFF Parameters to Tune Flexural Properties

To evaluate the material properties, a comparison was made between the flexural strength, the flexural modulus, and the failure mode of PP- and PA-based printed parts. This failure mode strongly differed for cCF-PP and cCF-PA12, consistent with the already discussed wetting results. The cCF-PA12 part was always totally fractured after delamination or partially cracked (Figure S10B in the Supplementary Materials), unlike the cCF-PP samples, which never broke as a whole (Figure S10A in the Supplementary Materials).

A typical flexural curve of a cCF-PP sample exhibits, first, a linear slope, and then multiple smaller stress drops, followed by a larger drop, as shown in Figure S10A in the Supplementary Materials. The small drops can be attributed to partial delamination and breakage of fiber bundles. During the last drop, the remaining polymer broke but the specimens were still held together by an outer PP layer and a cCF intact bundle. The flexural test was manually stopped at one point as the specimens never broke completely, and the stress level remained constant. The convex side of the sample, i.e., the side in which the tensile stress is created, showed PP stress whitening. There are two potential explanations for discoloration, strain-induced crystallization or formation of cavities [57]. For more insights, the DSC test was conducted on samples from a broken area and samples that did not undergo the flexural test to assess the degree of crystallinity using the following equation [58]:

(7) $$Xc\left(\%\right)=\frac{{∆H}_{m}-{∆H}_{cc}}{\Delta H_{m}^{\infty }.w}\times 100\%\;$$

where ${∆H}_{m}$ is the melting enthalpy, ${∆H}_{cc}$ is the cold crystallization enthalpy, $\Delta H_{m}^{\infty }\;$ is the melting enthalpy, and *w* is the weight fraction of the sample in grams.

The results indicated a significant reduction in crystallinity, dropping from 23.7% to 6.69% for the samples that were not subjected to the flexural test and those from the broken area, respectively. This suggests that the likely cause of the observed stress whitening is the formation of cavities (see Figures S11 and S12 in the Supplementary Materials).

Figure S10B in the Supplementary Materials displays two typical flexural curves of cCF-PA12 samples. The dotted curve shows a typical first type of failure mode, which indicates that the composite breaks as one homogeneous material. This mode of failure suggests that the interface between the CF and the PA12 polymer matrix is optimal. The second failure mode, which is displayed as a full line in Figure S10B, exhibits several drops in flexural stress after the linear increase. Unlike the failure mode in cCF-PP parts, the final drop of stress in the second failure mode of cCF-PA parts indicates a complete fracture of the specimen. It should be noted that only in rare cases did cCF-PA12 samples not show the second type of failure mode.

To obtain a deeper insight into the relation between printing conditions and the material properties of the samples, the response surface analysis approach was additionally applied [59]. The experimental data that were used to construct the response surfaces can be found in the Supplementary Materials. Table S5 in the Supplementary Materials summarizes the flexural measurement results, considering both the flexural modulus ($E_{flex}$) and the flexural strength ($\sigma_{flex}$), while Figures S16–S22 in the Supplementary Materials show the strength–strain curves for the measured samples and the bar charts used to analyze the results.

**Figure 7:**
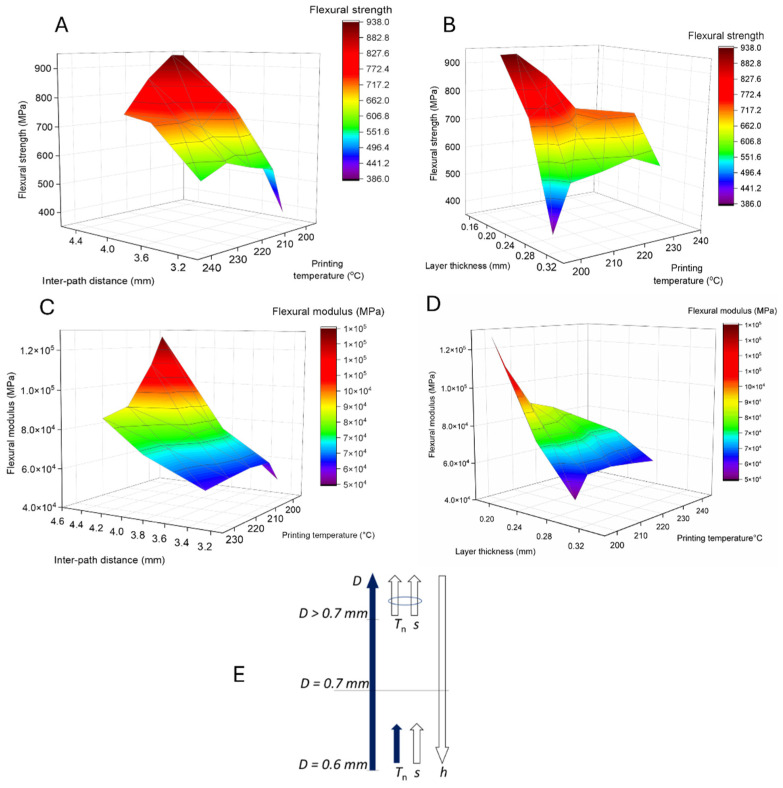
For the PA-based composite, response surface plots of the relationship between the (**A**) flexural strength ($\sigma_{flex}$), interpath distance, and printing temperature; (**B**) flexural strength ($\sigma_{flex}$), layer thickness, and printing temperature; (**C**) $E_{flex}$ modulus, interpath distance, and printing temperature; and (**D**) $E_{flex}$ modulus, layer thickness, and printing temperature of the samples. Specimens from F016 and F017, printing conditions P184–P215, with a *D* of 0.6 mm (Table S8). (**E**) Schematic representation of how the filament and printing parameters influence the flexural properties: the direction of the arrows indicates an increase/decrease in the printing parameter; the color reflects a positive (white) or negative (dark blue) impact on the flexural properties, and the blue ellipse highlights that the combination of the parameters has an influence.

Selecting a *D* of 0.6 mm, Figure 7 shows for the PA-based composite the response surfaces for $\sigma_{flex}$ and $E_{flex}$ depending on *s*, *h*, and the printing temperature (*T_n_*). A major influence of the printing temperature can be observed in Figure 7A on $\sigma_{flex}$, due to the need for sufficient melting of the polymer and thus better interlayer adhesion. It is interesting to note that the best flexural strength results were observed when the printing temperature was at the lower limit for a smaller *D*, which evidently minimizes the degradation of the polymer material. Increasing the value of *s* from 3.35 to 4.4 mm has a notable effect on the material properties, as shown in Figure 7A. A synergy is even observed when the material is printed at lower temperatures and a higher *s*, as the (molten) filament has enough room to flow during melting, resulting in smaller voids and enhanced material properties.

Figure 7B further shows that *h* significantly contributes to the $\sigma_{flex}$ of the printed parts. $\sigma_{flex}$ increases more than two times, from ca. 400 MPa to 900 MPa, when *h* decreases from ca. 0.3 mm to ca. 0.2 mm. However, the influence of temperature is more complicated in comparison with Figure 7A. A positive effect on $\sigma_{flex}$ is observed with a higher temperature, but the best results are only obtained when *h* and the temperature are smallest. In general, a small *h* ensures more efficient melting and interlayer adhesion, even at the lowest printing temperature, whereas a higher temperature may induce polymer degradation and overflow of the printing stands, as schematically shown in Figure 6C, leading to poor material properties.

The dependence of $E_{flex}$ on temperature and *s* (Figure 7C), as well as on temperature and *h* (Figure 7D), follows the same trend as for $\sigma_{flex}$, already discussed in Figure 7A,B. In particular, the highest $E_{flex}$ is obtained when the part is printed at the lowest temperature with the highest *s* (Figure 7C). It should be noted that, although the dependence is more pronounced, the modulus values decrease more rapidly if *s* deviates from the maximum and the temperature increases. In particular, $E_{flex}$ decreases by 35% upon going from the lowest printing temperature to the highest, whereas only a 20% variation upon changing the printing temperature was observed for $\sigma_{flex}$ (Figure 7A). A similar evolution of modulus vs. printing temperature can be observed when *h* changes (Figure 7D). The synergy of a low printing temperature and sufficient *s* is thus more important for the modulus value than for the strength.

A similar dependence of the flexural properties on the temperature and *h* was observed for the filament with *D* = 0.7 mm (instead of *D* = 0.6 mm), still focusing on the PA-based composite, as shown in Figure S13 in the Supplementary Materials. However, it is interesting to note that for the filaments with an even higher *D* = 0.9 mm, the influence of the temperature becomes more crucial. As Figure S14 in the Supplementary Materials shows, only the highest printing temperature leads to the best material performance independently of *s* and *h*. Evidently, a large filament diameter and a higher fraction of polymer (a higher diameter and a lower fraction of cCF) require more energy to enable sufficient melting, resulting in a greater influence on the printing temperature.

Figure 7E summarizes conceptually the influence of *s*, *h*, and printing temperature (*T_n_*) on the flexural properties (assuming a good wetting, as for the PA-based composite), with the direction of the arrows indicating an increase/decrease in the respective printing parameter, whereas the color variation reflects the positive/negative influence on the flexural properties.

### 3.4. Effect of Plasma Treatment and Annealing

As observed from the SEM images, the interfacial interactions of the cCF and PP matrix are poor. Hence, the attempt to improve the flexural properties was initiated with plasma treatment of the cCF before pre-impregnation. The results of the flexural tests of CF-PP with plasma-treated CF are summarized in Figure S16 in the Supplementary Materials. No significant difference can, however, be observed between $E_{flex}$ and $\sigma_{flex}$ for the sample prepared with the plasma treatment (printing conditions P093 and P095, Table S8 in the Supplementary Materials) and the non-treated cCF sample (printing condition P004, Table S8 in the Supplementary Materials). Thus, the main factor hindering the efficient wetting of the cCF with the PP matrix can be considered to be the high melting viscosity of the PP matrix.

The printed samples were alternatively annealed to close the voids and enhance the flexural properties. Prior to annealing, DSC analysis was performed to select the annealing conditions for PP and PA12, as the annealing temperature for semi-crystalline polymers should be selected between *T_g_* and *T_m_*, and ideally it should be close to *T_cc_*. To exclude the negative influence of the possible crystallinity increase after annealing, the DSC data were compared before and after annealing for both polymer matrix materials, as shown in Figures S24–S29 in the Supplementary Materials. The results show that the crystallinity percentage and the shape of the melting peak of the annealed parts are almost the same as those of the unannealed ones.

For the recycled PP (Figure S24 in the Supplementary Materials), no exothermal cold crystallization peak was observed during the first and the second heating DSC cycle. The small exothermal peak at approximately 120 °C in the heating stage was possibly due to a small contamination by (low-density) polyethylene ((LD)PE). The melting temperature was *T_m_* = 167 °C. Consequently, an annealing temperature should be selected between *T_g_* = −20 °C and this melting temperature. Three temperatures were selected, namely, 90 °C, 120 °C, and 140 °C, for the annealing of PP samples. The DSC curve of PA12 (the right-hand side of Figure S24 in the Supplementary Materials), by contrast, shows in its first run a cold-crystallization peak at around 165 °C. In this context, 140 °C and 165 °C were selected to anneal the cCF-PA12 parts.

**Figure 8:**
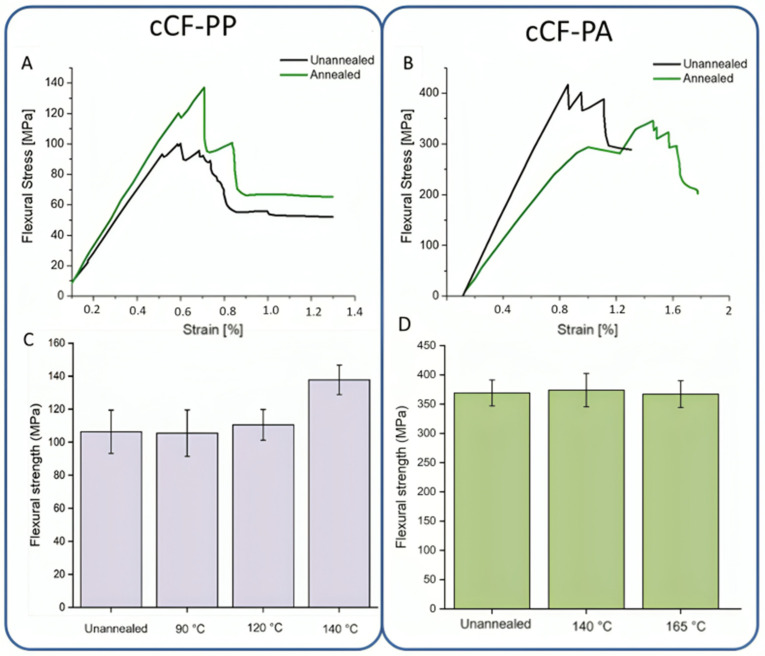
Representative flexural curves of (**A**) cCF-PP parts unannealed and annealed at 140 °C (specimen F004, printing conditions P002 and P088, respectively) and (**B**) cCF-PA12 parts unannealed and annealed at 165 °C (specimen F007, printing condition P107; specimen F009, printing condition P114, respectively). Effect of annealing at different temperatures on (**C**) cCF-PP parts at 90 °C, 120 °C, and 140 °C and (**D**) cCF-PA12 parts at 140 °C and 165 °C.

Analysis of the flexural curves shows that the annealed cCF-PP exhibits a slight change-over in the failure mechanism, as shown in Figure 8A. The annealed samples showed larger and more uniform drops in stress levels compared to the unannealed samples, which showed multiple smaller drops. Such a flexural curve profile is typical in case of increased interlayer strength and/or increased strength between the paths, which is achieved due to (partial) void closure. ImageJ analysis results demonstrated that the void volume in an annealed sample at 140 °C is reduced compared to the unannealed sample, as illustrated in Figure S32 in the Supplementary Materials. The small drops in the flexural strength ($\sigma_{flex}$) of the unannealed samples are due to more local delamination or fractures, whereas the layers of the annealed material are more uniformly delaminated.

Note that a change-over of failure mode for the cCF-PP could result in better interlayer bonding in the annealed samples. No significant increase in flexural strength ($\sigma_{flex}$), however, was noticed for the annealed specimens at 90 °C and 120 °C (Figure 8C). In contrast, at 140 °C, which allows for PP melting, an increase of ca. 30% in the flexural strength ($\sigma_{flex}$) was noticed compared to the unannealed samples and the samples that were annealed at lower temperatures. The flexural modulus ($E_{flex}$), however, remained unaffected (Figure 8C). Hence, only a careful selection of the annealing conditions allowed us to improve the mechanical properties of the cCF-PP parts.

For cCF-PA, no changes in the failure mode were observed, as the annealed parts broke similarly to the unannealed parts (Figure 8B). This suggests that the interlaminar strength did not improve significantly with the annealing process. Indeed, no increase in flexural strength ($\sigma_{flex}$) was observed for cCF-PA12 (Figure 8D) after annealing at all the selected temperatures. This result implies that the printed (unannealed) cCF-PA12 already exhibited optimal mechanical properties, and additional thermal treatment could even degrade PA12. Figure S33 in the Supplementary Materials displays unannealed and annealed samples of cCF-PA12 exhibiting effective closure of voids. Annealing is therefore likely unnecessary for improving the mechanical properties of the cCF-PA12 composite. In other words, annealing seems to be only necessary when the wetting is less.

## 4. Conclusions

This research focused on the manufacturing of polymer-based composites reinforced with continuous carbon fibers for utilization in 3D-printing applications. Initially, 17 filament formulations were developed using different matrix materials (recycled polypropylene and virgin polyamide 12) via a prepregnation process. Subsequently, bending bars were 3D-printed via FFF using the prepared prepreg filaments.

The study examined the influence of various 3D printing parameters, including the layer height (*h*), interpath distance (*s*), printing temperature (*T_n_*), and nozzle diameter of the impregnation mold (*D*), on the void formation and the flexural properties of the printed components. Explorations involved a range of *h* values from 0.19 to 0.55 mm, *s* values from 0.58 to 1.67 mm, *T_n_* values from 200 to 235 °C, and *D* values from 0.6 to 1.1 mm. Additionally, plasma treatment of cCF was assessed, and optimization of prepregnation parameters, such as cCF fractions ranging from 11.27% to 37.60%, was performed.

The cCF-PP specimens exhibited poor wetting characteristics. Analysis of the estimated shear rate (γ˙w) demonstrated low values, suggesting that the inadequate wetting observed in cCF-PP specimens can be attributed to high viscosity levels. Moreover, the void analysis indicated a slight reduction of 8.16% in void content for samples printed at higher temperatures of 235 °C compared to those printed at 225 °C. This decline is associated with reduced viscosity, facilitating improved wetting between the matrix and the filler components.

DSC results of cCF-PP specimens exhibited a substantial reduction in crystallinity of 72%. This reduction indicates stress whitening, attributed to cavitation formation. However, plasma treatment showed no significant difference and did not result in improvement. On the contrary, the cCF-PA specimens exhibited proper wetting of the fibers by the matrix, resulting in superior properties.

Both a high printing temperature (*T_n_*) and a larger interpath distance (*s*) had a positive impact on flexural properties, especially when combined with larger nozzle diameters (*D*), leading to a notable 20% increase in flexural strength. However, specimens with a high cCF fraction of 37.60% and a smaller nozzle diameter of 0.6 mm experienced negative effects from high temperatures, where a synergistic effect occurred with higher interpath distances and lower printing temperatures. In any case, reducing the layer height (*h*) from 0.3 mm to 0.2 mm resulted in a significant enhancement in flexural strength, demonstrating a remarkable increase of 125%.

In summary, this research offers guidelines for enhancing the flexural properties of cCF-based parts. For instance, annealing at sufficiently high temperatures proves particularly beneficial for PP-based composites, potentially increasing flexural strength by up to 30%. This strategy compensates for the reduced wetting observed in PP-based composites due to higher viscosity. Contrarily, for PA-based composites, wetting does not need to be further optimized and the focus can be on the optimal printing settings alone.

## Figures and Tables

**Table 1:** Parameters of the filament production from polypropylene PP108MF10 (PP), polyamide Rilsamid AMN O TLD (PA12), and Torayca T300B-3000 continuous carbon fiber (cCF) potentially treated with plasma. $V_{f}$—fiber volume fraction; *V_pultrusion_*—pultrusion speed; *T_S_*_1_ and *T_S_*_2_—extruder screw temperature; *T_C_*—temperature of impregnation chamber; *T_P_*_1_, *T_P_*_2_, and *T_P_*_3_—temperature of impregnation pins; *D*—nozzle diameter; and *N*—rotational speed of the extruder.

Matrix	Filament	$V_{f}$ (%)	*V_pultrusion_* [mm/min]	*T_S_*_1_ [°C]	*T_S_*_2_ [°C]	*T_C_* [°C]	*T_P_*_1_ [°C]	*T_P_*_2_ [°C]	*T_P_*_3_ [°C]	*D* [mm]	*N* [rpm]
PP	F001	16.43	300	180	230	230	230	230	230	1.1	0.68–0.8
	F002 ^1^	14.84	300	180	230	230	230	230	230	1.1	2.05
	F003 ^2^	15.23	300	180	230	230	230	230	230	1.1	2.05–2.2
	F004	16.37	300	180	230	230	230	230	230	1.1	2.05–2.2
	F005	11.27	300	185	225	215	240	240	240	1.1	0.65
PA	F006	13.50	300	185	225	225	240	240	240	1.1	1.15
	F007	12.63	300	185	225	225	240	240	240	1.1	1.1
	F008	14.91	300	185	225	225	240	240	240	1.1	1.1
	F009	14.73	550	185	225	225	240	240	240	1.1	2
	F010	14.02	425	185	225	225	240	240	240	1.1	1.8
	F011	14.39	675	185	225	225	240	240	240	1.1	2.3
	F012	14.33	800	185	225	225	240	240	240	1.1	2.3
	F013	18.70	300	185	225	225	240	240	240	0.9	0.95
	F014	30	300	185	225	225	240	240	240	0.7	0.47
	F015	30	300	185	225	225	240	240	240	0.7	0.47
	F016	37.60	300	185	225	225	240	240	240	0.6	0.35
	F017	37.60	300	185	225	225	240	240	240	0.6	0.35

^1^ Carbon fiber was treated with O_2_ + He plasma. ^2^ Carbon fiber was treated with Ar + He plasma.

**Table 2:** Layer height (*h*) and interpath distances (theoretical *s* and actual *s*, *s_actual_*) of printed parts, starting from 4 filaments with specifications in Table 1.

*D* (mm)	Filament	Sample	*T_n_* (°C)	*h* (mm)	*s* (mm)	*s_actual_* (3 × *s_actual_*) (mm)
1.1	F001	P002	215–235	0.35	1.67	7.02
	F001	P004	215–235	0.45	1.30	5.46
	F001	P005	215–235	0.55	1.06	4.46
0.9	F013	P126	215–235	0.29	1.37	5.50
	F013	P135	215–235	0.37	1.06	4.50
	F013	P145	215–235	0.45	0.87	3.85
0.7	F014	P154	205–235	0.22	1.06	4.85
	F014	P164	205–235	0.29	0.83	4.10
	F014	P172	205–235	0.35	0.68	3.75
0.6	F016	P184	200–225	0.19	0.91	4.40
	F017	P185	200–225	0.25	0.71	3.80
	F017	P195	200–225	0.30	0.58	3.35

**Table 3:** Printing parameters of untreated and treated CF with PP. Specification of original filaments in Table 2.

Filament	Printing Conditions	cCF	*h* (mm)	*s* (mm)	*T_n_* (°C)	*T_bed_* (°C)
F001	P004	untreated	0.45	1.30	230	60
F002	P093	He/O_2_	0.45	1.30	235	80
F003	P095	He/Ar	0.45	1.30	235	80

**Table 4:** The annealing and printing parameters. Specification of original filaments in Table 1.

Matrix	Printing Conditions	Filament	*D* (mm)	*h* (mm)	*s* (mm)	*T_n_* (°C)	*T_b_* (°C)	Annealing Temperature (°C)
PP	P088	F004	0.9	0.45	1.30	235	80	90, 120, and 140
	P089							
	P092							
PA12	P113–P115	F009	0.9	0.35	1.67	215	110	140, and 165
	P122–P125	F010						
	P116–P119	F011						
	P120–P121	F012						

## Data Availability

Data are contained within the article and Supplementary Materials.

## Supplementary Materials

The following supporting information can be downloaded at: https://www.mdpi.com/article/10.3390/ma17081788/s1, Figure S1: Cross-section of a spreader pin and fiber bundle (dashed line) forces; Table S1: The properties of the cCF and the polymers matrices; Table S2: Dimensions of ISO-14125 flexural specimens (ISO-14125); Figure S2: SEM results of PP composite obtained from printing F001: (a) compression fracture, (b) delamination zone, (c) poor interfacial interaction between the matrix and the fiber, and (d) broken fibers inside the matrix; Figure S3: SEM results for PA12 composite obtained from F013: (a) CF dispersion in the matrix PA12, (b) compressive fracture for composites, (c) interfacial interaction between the matrix and the fiber, and (d) individual fiber that is fully coated with PA12; Figure S4: Cross-section of the annealed sample made with CF-PA12; Figure S5: Cross-section of the annealed sample made with CF-PP; Figure S6: Viscosity vs. shear rate temperature dependence for (a) PA12 and (b) PP; Table S3: The estimated shear rate for PP during the printing process; Table S4: The results of the void analysis of PP printed samples; theoretical support for Equation (6) in the main text; Figure S7: (A) Deformation of the printing filament during the FFF process. (B) Cross-section of the printed strand; Figure S8: (A) The effect of partially overlapping composite tracks, where *s* ≪ *w* and *ξ* ≈ 1. The order of strand deposition is indicated by increasing numbers. Cross-section of a single-layer composite perpendicular to the fiber direction. (B) Reduction in the macro-void content by slightly overlapping the composite tracks, where *ξ* = 0.5. Cross-section of a triple-layer composite perpendicular to the fiber direction; Figure S9: The relationship between *h* and the voids showcases a comparison between calculated and actual values; Figure S10: Averaged flexural stress curves for (A) cCF-PP (specimen F001, printing condition P002; Table S8) and (B) cCF-PA12 (specimens F007 and F009, printing conditions P107–P113; Table S8); Figure S11: DSC results for PP-cCF samples taken from a broken area; Figure S12: DSC results for PP-cCF samples taken from samples that did not undergo the flexural test; Figure S13: Response surface graphs of the relationship between the (A) flexural strength ($\sigma_{flex}$), interpath distance, and printing temperature; (B) flexural strength ($\sigma_{flex}$), layer thickness, and printing temperature; (C) $E_{flex}$ modulus, interpath distance, and printing temperature; and (D) $E_{flex}$ modulus, layer thickness, and printing temperature of the samples (specimen F014, printing conditions P154–P183, with *D* = 0.7 mm); Figure S14: Response surface graphs of the relationship between (A) flexural strength ($\sigma_{flex}$), interpath distance, and printing temperature; (B) flexural strength ($\sigma_{flex}$), layer thickness, and printing temperature; (C) $E_{flex}$ modulus, interpath distance, and printing temperature; and (D) $E_{flex}$ modulus, layer thickness, and printing temperature of the samples (specimen F013, printing conditions P126–P153, with *D* = 0.9 mm); Figure S15: Response surface graphs of the relationship between (A) flexural strength ($\sigma_{flex}$), interpath distance, and printing temperature; (B) flexural strength ($\sigma_{flex}$), layer thickness, and printing temperature; (C) $E_{flex}$ modulus, interpath distance, and printing temperature; and (D) $E_{flex}$ modulus, layer thickness, and printing temperature of the samples (specimen F001, printing conditions P001–P066, with *D* = 1.1 mm); Figure S16: Flexural strength ($\sigma_{flex}$) and flexural modulus ($E_{flex}$) of plasma-treated and untreated cCF-PP (specimens F001, F002, and F003). The printing conditions are highlighted in the plots; Figure S17: The sample failure mode of F013, printing condition P126, with *D* = 0.9 mm (PA12 as a matrix); Figure S18: The sample failure mode of F014, printing condition P164, with *D* = 0.7 mm (PA12 as a matrix); Figure S19: The sample failure mode of F017, printing condition P190, with *D* = 0.6 mm (PA12 as a matrix); Figure S20: Flexural strength and modulus of the samples with filament diameter *D* = 1.1 mm; Figure S21: Flexural strength and modulus of the samples with *D* = 0.9 mm; Figure S22: Flexural strength and modulus of the samples with *D* = 0.7 mm; Figure S23: Flexural strength and modulus of the samples with *D* = 0.6 mm; Table S5: Flexural strength and modulus of the samples with different *D*, *T*, *s*, and *h* values; Table S6: DSC test parameters; Figure S24: DSC results for PP; Figure S25: DSC results for PA12; Figure S26: DSC results for unannealed cCF-PP; Figure S27: DSC results for annealed cCF-PP; Figure S28: DSC results for unannealed cCF-PA12; Figure S29: DSC results for annealed cCF-PA12; Figure S30: TGA results for PP; Figure S31: TGA results for PA; Table S7: Estimated flexural properties of the cCF composites for varying fiber fractions; Figure S32: ImageJ results for (A) a non-annealed cCF-PP (P62) sample and (B) an annealed cCF-PP sample at 140 °C; Figure S33: ImageJ results for (A) a non-annealed cCF-PA12 (P142) sample and (B) an annealed cCF-PA12 sample at 165 °C; Table S8: Printing conditions for the test specimens.
