# Supplementary material for: Designing Prepregnation and Fused Filament Fabrication Parameters for Recycled PP- and PA-Based Continuous Carbon Fiber Composites
Source: Materials (Basel). 2024 Apr 12;17(8):1788. doi: 10.3390/ma17081788 (PMC11050926; doi:10.3390/ma17081788)
Supplement: Supplementary file 1 [file materials-17-01788-s001.zip › materials-2938336-supplementary.pdf]

Supporting Information for

Designing prepregnation and fused filament fabrication parameters for recycled PP- and PA-based-continuous carbon fiber composites

Marah Baddour[1], Ruth Garcia-Campà[2], Pablo Reyes,[1,3] Dagmar R. D'hooge [3,4], Ludwig Cardon[1], and Mariya Edeleva[1]

[1] Centre for Polymer and Material Technologies, Department of Materials, Textiles and Chemical Engineering, Ghent University, Technologiepark 130, 9052 Zwijnaarde, Belgium;
[2] Leitat Technological Center, C/Innovacio 2, 08225 Terrassa, Barcelona, Spain
[3] Laboratory for Chemical Technology, Department of Materials, Textiles, and Chemical Engineering, Ghent University, Technologiepark 125, 9052 Zwijnaarde, Belgium;
[4] Centre for Textile Science and Engineering, Department of Materials, Textiles, and Chemical Engineering, Ghent University, Technologiepark 70a, 9052 Zwijnaarde, Belgium;

Corresponding author: mariya.edeleva@ugent.be

General information

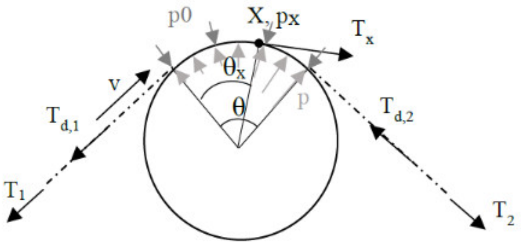

Figure S1. Cross-section of a spreader pin and fiber bundle (dashed line) forces.

Table S1. The properties of the cCF and the polymers matrices.

Material	Tensile strength (MPa)	Young Modulus (GPa)	Yield strain (%)	Flexural strength (MPa)	Density (g/cm³)	T_m (°C)
**PP**	19*	1	8	17	0.90	164
**PA12**	42*	1.45	7	45	1.02	178
**T300B-3000**	3530	230	1.26	(3530)**	1.76	/

** These values are the yield stress of the PP & PA12 reported by the materials datasheet as the ultimate tensile stress was not given. ** Tensile strength from the datasheet is reported.*

Table S2. Dimensions of ISO-14125 flexural specimens (ISO-14125).

Method	Length *l* (mm)	Width *b* (mm)	Thickness *h* (mm)	Distance between supports *L* (mm)	Distance between loads *L′* (mm)
**Method A, Class IV**	100	15	2	80	/
**Method B, Class IV**	100	15	2	81	27
**Tolerances**	+10 -0	± 0,5	± 0.2	± 1	± 1

Morphology analysis via SEM and optical microscopy

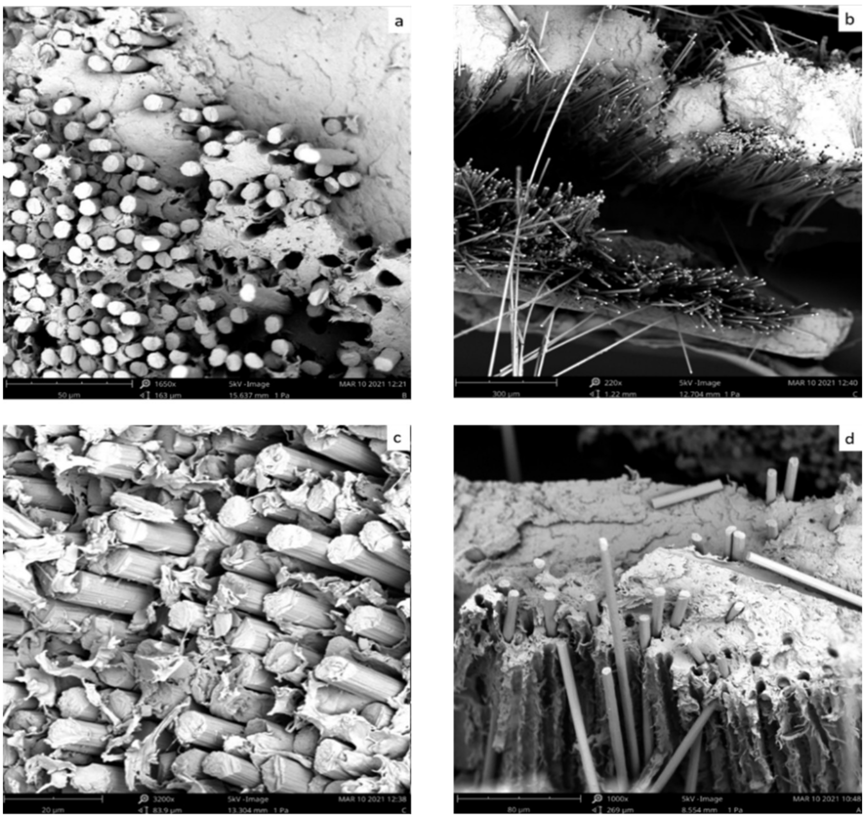

Figure S2. SEM results of PP composite obtained from printing F001. a) Compression fracture, b) Delamination zone, c) Poor interfacial interaction between the matrix and the fiber, and d) Broken fibers inside the matrix.

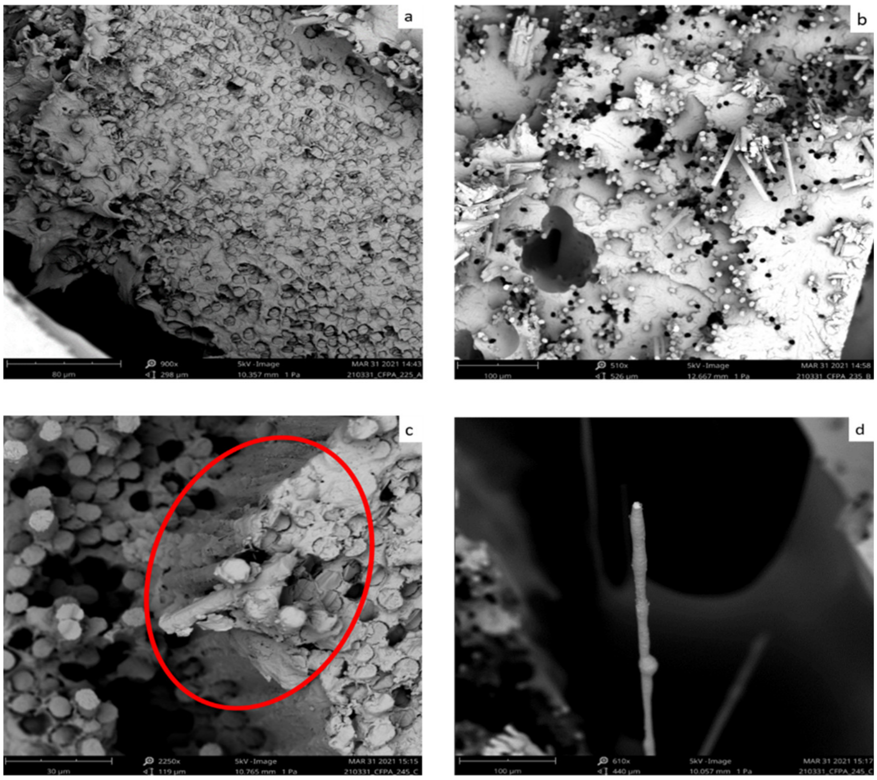

Figure S3. SEM results of PA12 composite obtained from F013. a) CF dispersion in the matrix PA12, b) Compressive fracture for composites, c) Interfacial interaction between the matrix and the fiber, and d) Individual fiber that is fully coated with PA12.

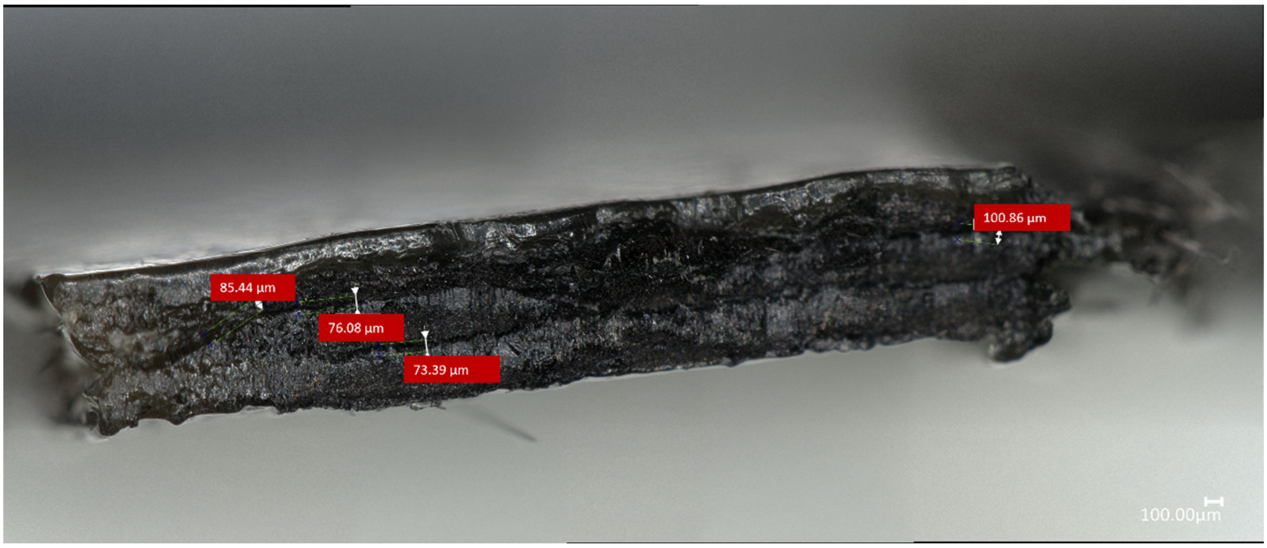

Figure S4. Cross section of the annealed sample made with CF-PA12.

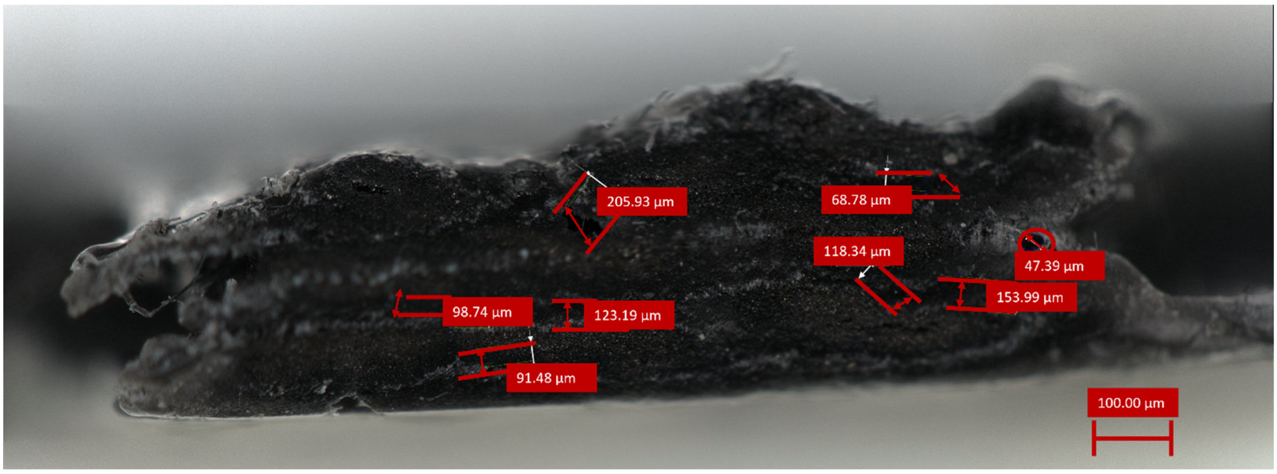

Figure S5. Cross section of the annealed sample made with CF-PP.

Rheological properties

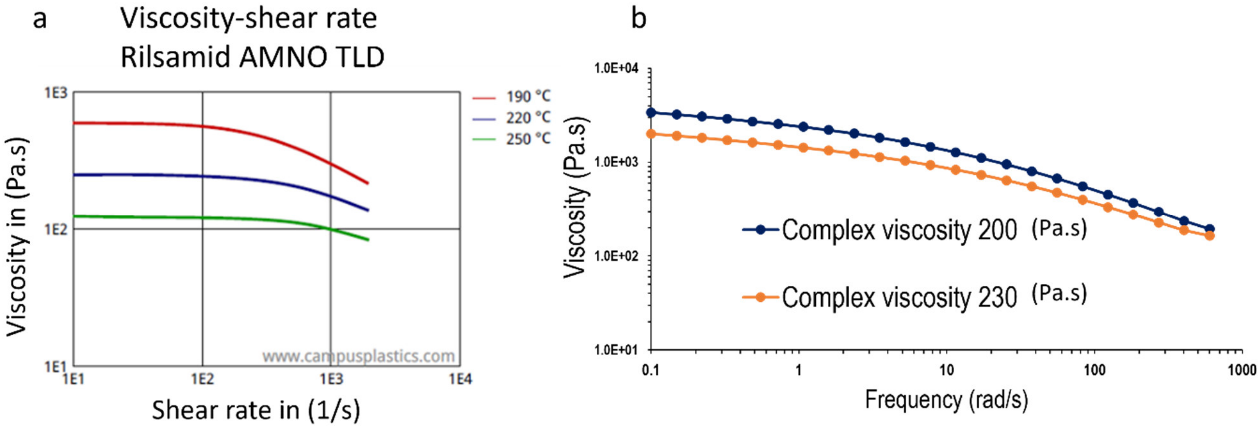

Figure S6. Viscosity vs. shear rate temperature dependence for a): PA12 matrix and b): PP matrix.

Table S3. The estimated shear rate for PP during the printing process.

T (°C)	Q (cm^3/s)	Melt density (g/cm^3)	$\dot{\gamma}a$ (s^{-1})	$\dot{\gamma}w$ (s^{-1})
215	0.02	0.50	17	34
225	0.01	0.73	17.04	34.08
235	0.01	0.74	16.89	33.78

Table S4. The results of the voids analysis of PP printed samples.

Sample code	count	Total area (μm²)	Average size (μm²)	Area (%)	Mean (μm)
P062	5454	78834.92	14.45	4.5	5.15
P076	6733	82975.77	12.32	4.9	6.285

Theoretical support for Equation (6) in the main text

During the deposition, a thermoplastic-based filament with a circular cross-section with diameter d_1 is fed through a heated nozzle with an internal diameter d_2, which is often smaller than d_1, and deposited on the printing platform. The orientation of the composite strand changes from normal to the deposition platform to parallel to the deposition platform (Figure S7 A). In the studied FFF process, the velocity v_1 of the composite in the nozzle should be equal to deposition velocity v_2 (Equation S1). The main reason for this is the high Young's modulus of a typical fiber material, which remains in the solid phase during processing.

$$v_1 = v_2 \qquad \text{Eq. S1.}$$

Furthermore, due to the deformability of the melted composite strand, the form changes from a circular cross-section to a rectangular one with semi-circular ends (Figure S7 B). The cross-sectional surface area of the strand can be calculated based on the geometrical scheme in Figure S7 B assuming the radius of the spherical ends being *r*, *h* – the layer thickness, and *w* – track width.

$$S_{stand} = w \times h - 4 \times \ r^2(1 - \tfrac{\pi}{4}) \qquad \text{Eq. S2}$$

The maximal value for *r* can be *h*/2 which gives:

$$S_{stand} = w \times h - h^2\left(1 - \tfrac{\pi}{4}\right) = h \times \left(w - h\left(1 - \tfrac{\pi}{4}\right)\right) = \text{"}height\text{"} \times \text{"}length\text{"} \qquad \text{Eq. S3}$$

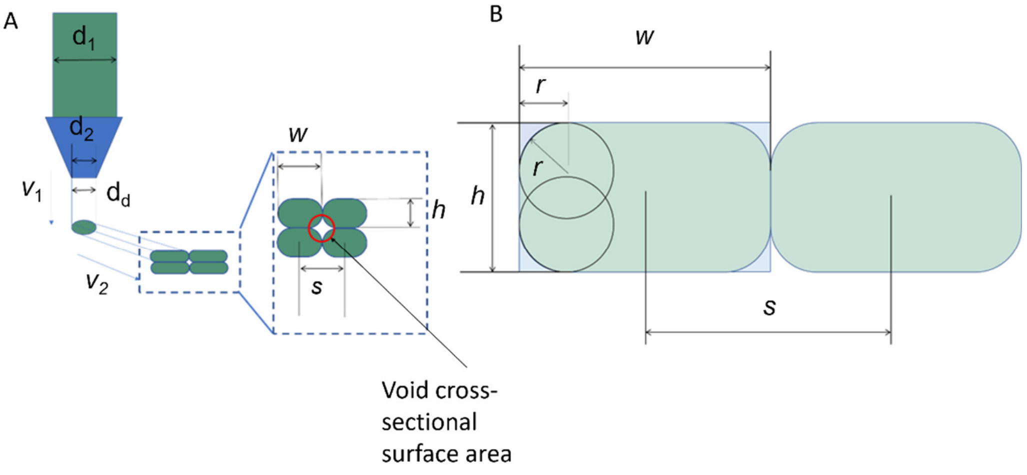

Figure S7. A: Deformation of the printing filament during the FFF process. B: Cross-section of the printed strand.

Based on Equation S1 and the law of mass conservation, it can be said that the composite strand's cross-sectional area is equal to the filament's cross-sectional area at a given temperature and time.

As a result, Equation S3 gives the relation between the diameter of the filament d_d, the height h, and width w of a deposited composite track. In practice, d_d and h are predefined, and the resulting track width is given in Equation S4. By changing the inter-path distance s, i.e. the distance between the centers of two adjacent and parallel composite tracks can be adapted.

$$\frac{\pi d_d^2}{4} = (w - h)h + \frac{\pi h^2}{4} \qquad \text{Eq. S4}$$

$$w = \frac{\pi d_d^2}{4h} + h\left(1 - \frac{\pi}{4}\right) \qquad \text{Eq. S5}$$

In the context of FFF, a tool path can be defined as the movement of an extruder/deposition nozzle relative to the build platform, which is necessary to build up a three-dimensional object. The tool path for the production of a unidirectional composite layer will consist of parallel tool paths with an inter-path distance s.

$$s = w \qquad \text{Eq. S6}$$

A value for s equal to w ensures that the individual tracks are making contact (Figure S7), but this inherently leads to a certain macroscopic void surface fraction $S_{v,mac}$ in between the individual deposited tracks, as highlighted in Figure S7 A with the red circle. $S_{v,mac}$ can be determined via Equation S7 which is derived via a simple geometrical sketch as shown in Figure S7 B. Note that $S_{v,mac}$ refers to the macroscopic voids in between composite tracks, not including the microscopic voids within an individual composite track after its deposition ($S_{v,mic}$) which is caused by material degradation.

$$S_{v,mac} = h \times h\left(1 - \frac{\pi}{4}\right) \qquad \text{Eq. S7}$$

In theory, it is possible to reduce or even eliminate macro-voids in a composite object by depositing slightly overlapping tracks, *i.e.* choosing $s < w$. In practice, to obtain an optimum between density and printability we can define a macro-void filling factor ξ between 0 and 1 which is determined experimentally (Equation S8).

$$s = w - \xi \cdot h \cdot (1 - \frac{\pi}{4}) \qquad \text{Eq. S8}$$

This equation is formed in the following way: we transform the "length" term from eq. S3 with an overlap macro-void filling factor. In this, $\xi = 0$ corresponds to $s = w$ and a $S_{v,mac}$ as calculated in Equation S7, while $\xi = 1$ corresponds to $s = s_{min}$ (Equation S8) and $S_{v,mac} = 0$. However, due to limitations in the deformability of the strands being deposited, a situation in which a track is partially deposited on top of another track in the same layer, instead of filling up the existing voids, can occur for high values of ξ (Figure S8 A). This effect leads to reduced printability and will induce fiber failure during deposition. In practice, an ξ-value of around 0.5 is realistic. In this case, the lower half of the rhombic macro-void cross-sections are filled making a trapezoid with semi-rounded ends as shown in Figure S7 B.

$$s_{min} = w - h\left(1 - \frac{\pi}{4}\right) = \frac{\pi d_d^2}{4h} \qquad \text{Eq. S9}$$

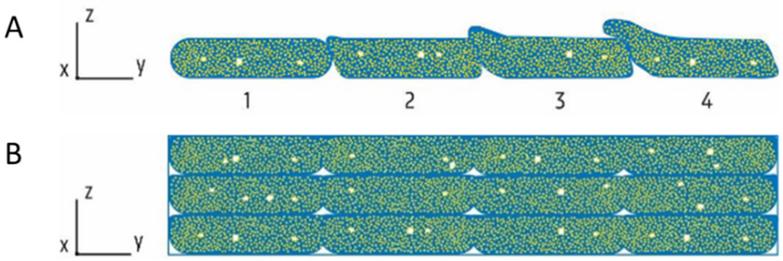

Figure S8. A: The effect of partially overlapping composite tracks, where $s \ll w$ and $\xi \approx 1$. The order of strand deposition is indicated by increasing numbers. Cross-section of a single-layer composite perpendicular to the fiber direction. B: Reduction of the macro-void content by slightly overlapping the composite tracks, where $\xi = 0.5$. Cross-section of a triple-layer composite perpendicular to the fiber direction.

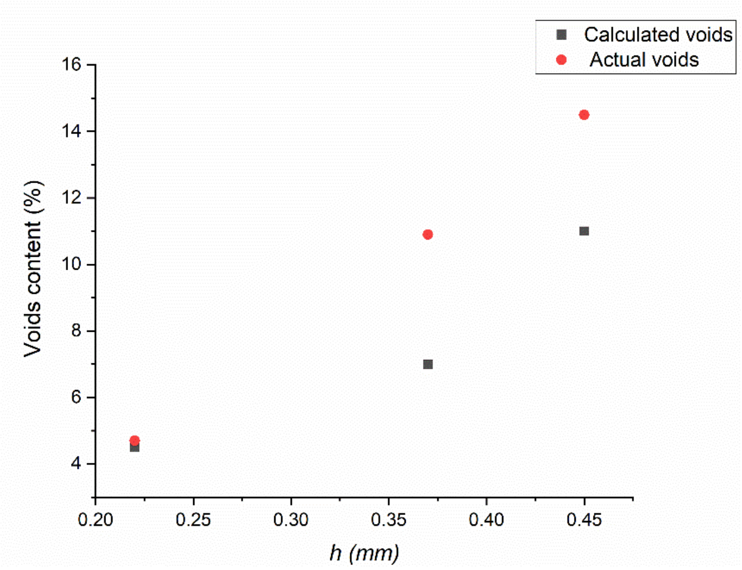

Figure S9. The relationship between h and the voids showcases a comparison between calculated and actual values.

Flexural testing

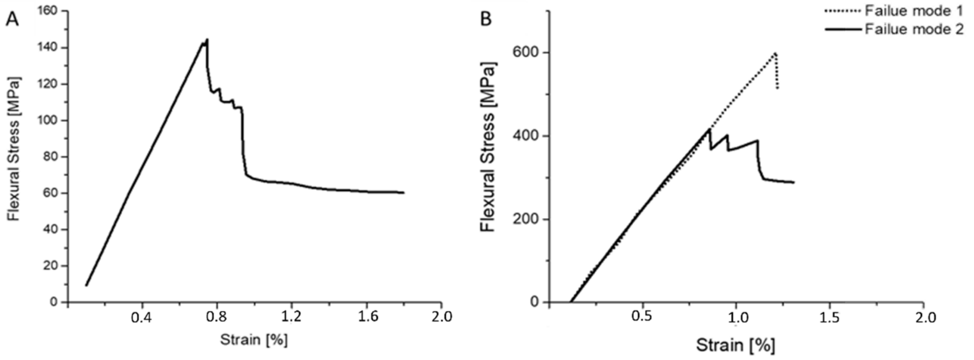

Figure S10. Averaged flexural stress curve of A: cCF-PP (Specimen F001, Printing conditions P002, Table S8), B: cCF-PA12 (Specimen F007 and F009, Printing conditions P107-P113, Table S8.).

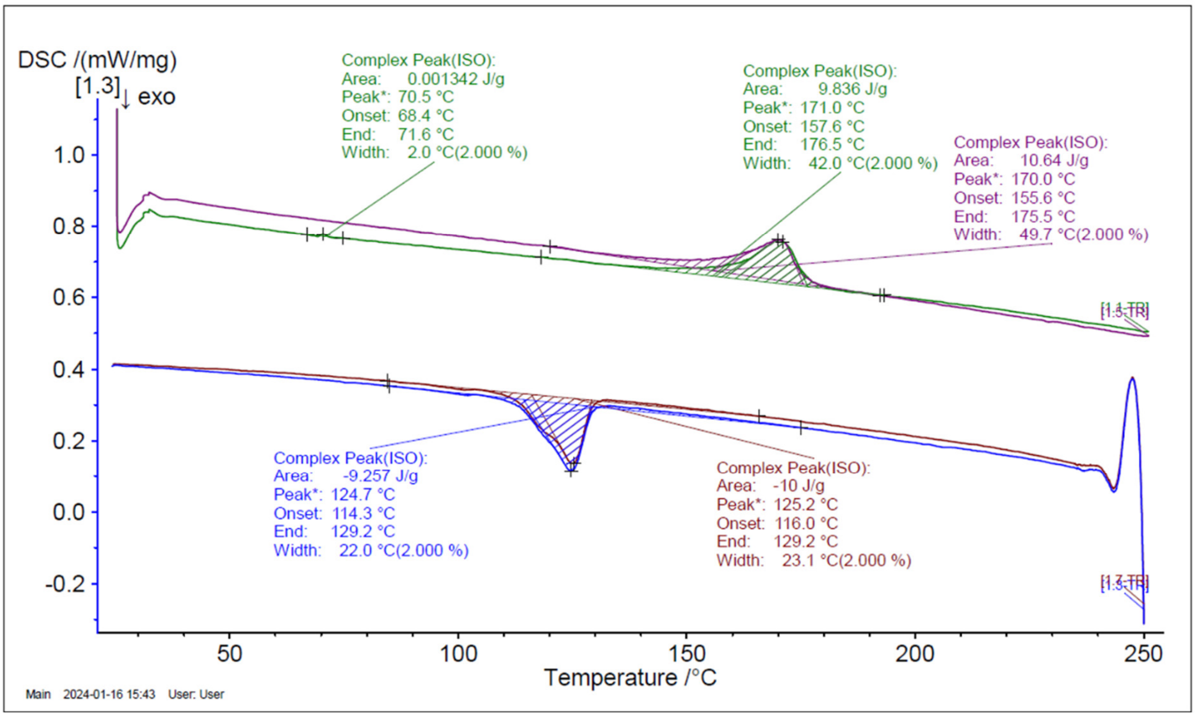

*Figure S11. DSC results for PP-cCF samples taken from a broken area (peak *:the location of the peak maximum).*

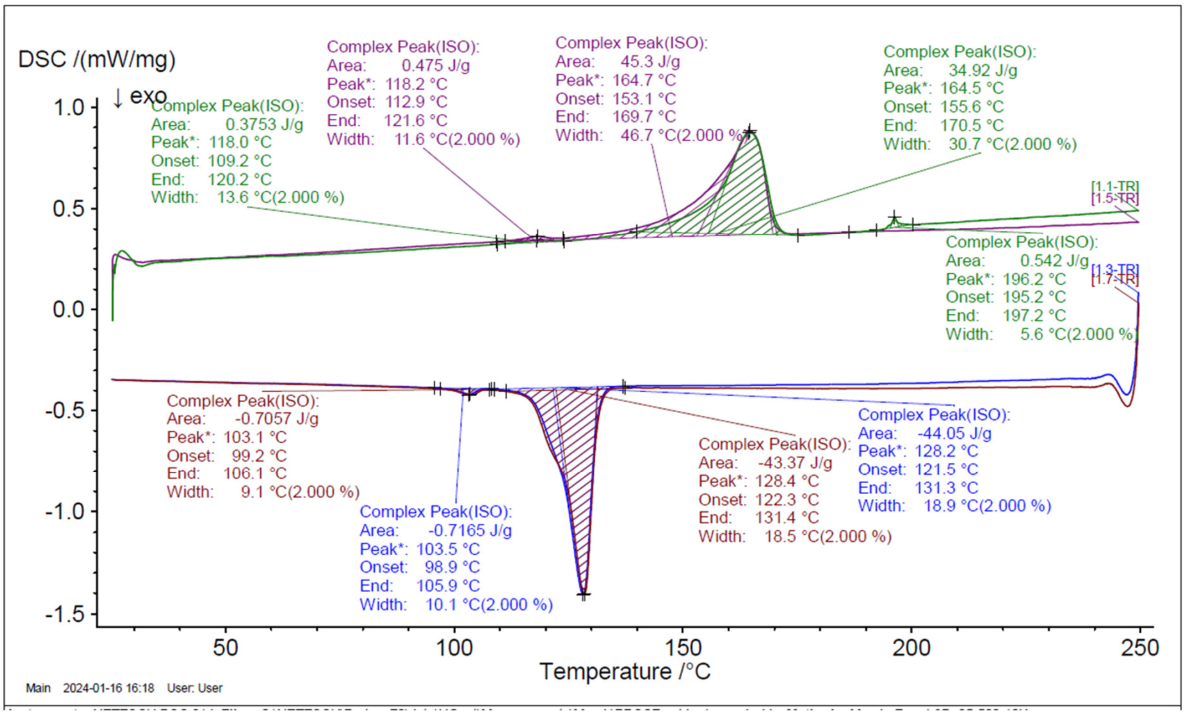

*Figure S12. DSC results for PP-cCF samples were taken from samples that did not undergo the flexural test (peak *:the location of the peak maximum).*

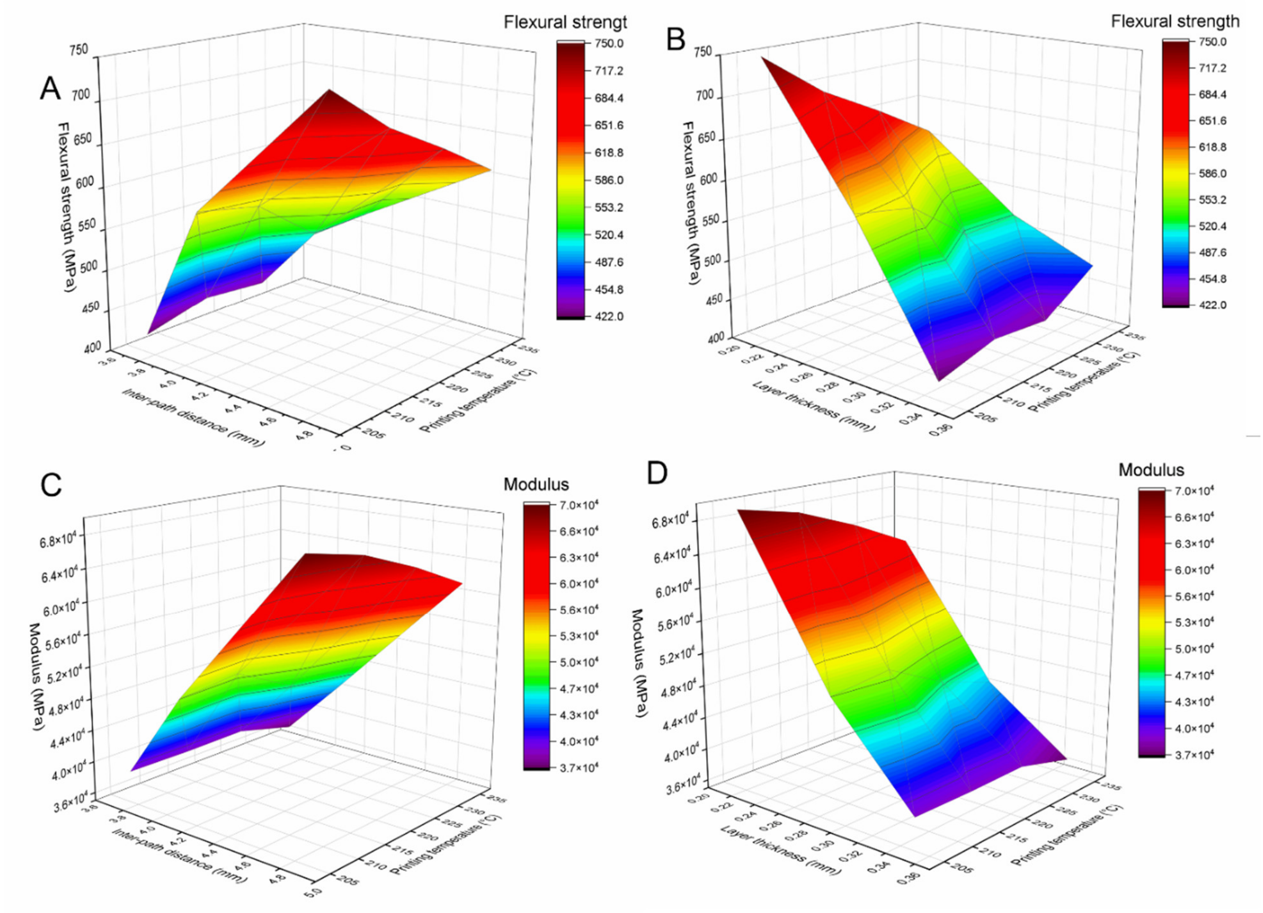

Figure S13. Response surface graphs of the relationship between A: Flexural strength σ_{flex}, inter-path distance, and printing temperature, and B: Flexural strength σ_{flex}, layer thickness, and printing temperature, C: Modulus E_{flex}, inter-path distance, and printing temperature, and D: Modulus E_{flex}, layer thickness, and printing temperature of the samples Specimens from F014 Printing conditions P154-P183 with D=0.7 mm (PA12 as a polymer matrix).

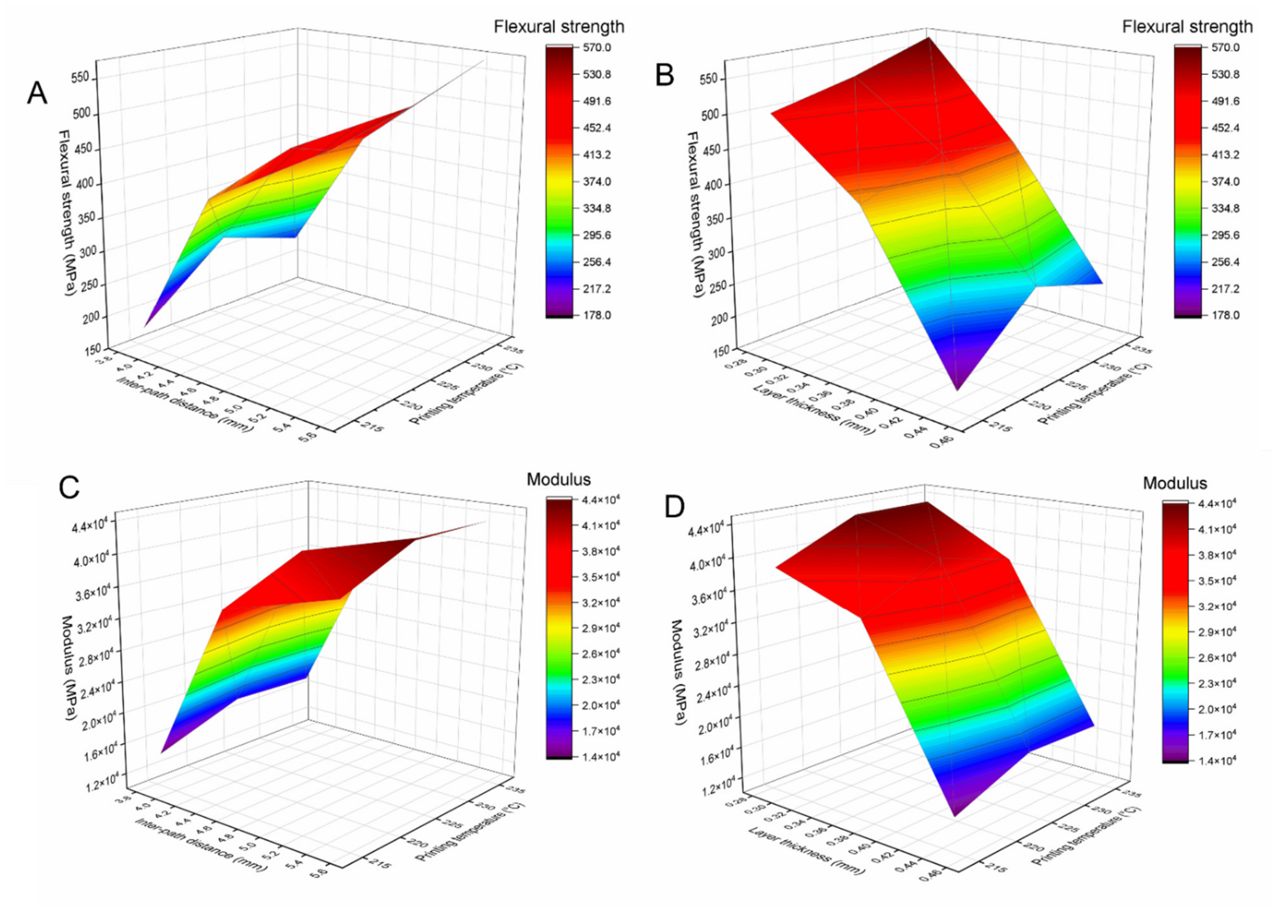

Figure S14. Response surface graphs of the relationship between A: Flexural strength σ_{flex}, inter-path distance, and printing temperature, and B: Flexural strength σ_{flex}, layer thickness, and printing temperature, C: Modulus E_{flex}, inter-path distance, and printing temperature, and D: Modulus E_{flex}, layer thickness, and printing temperature of the samples Specimens from F013 Printing conditions P126-P153 with D=0.9 mm (PA12 as a polymer matrix).

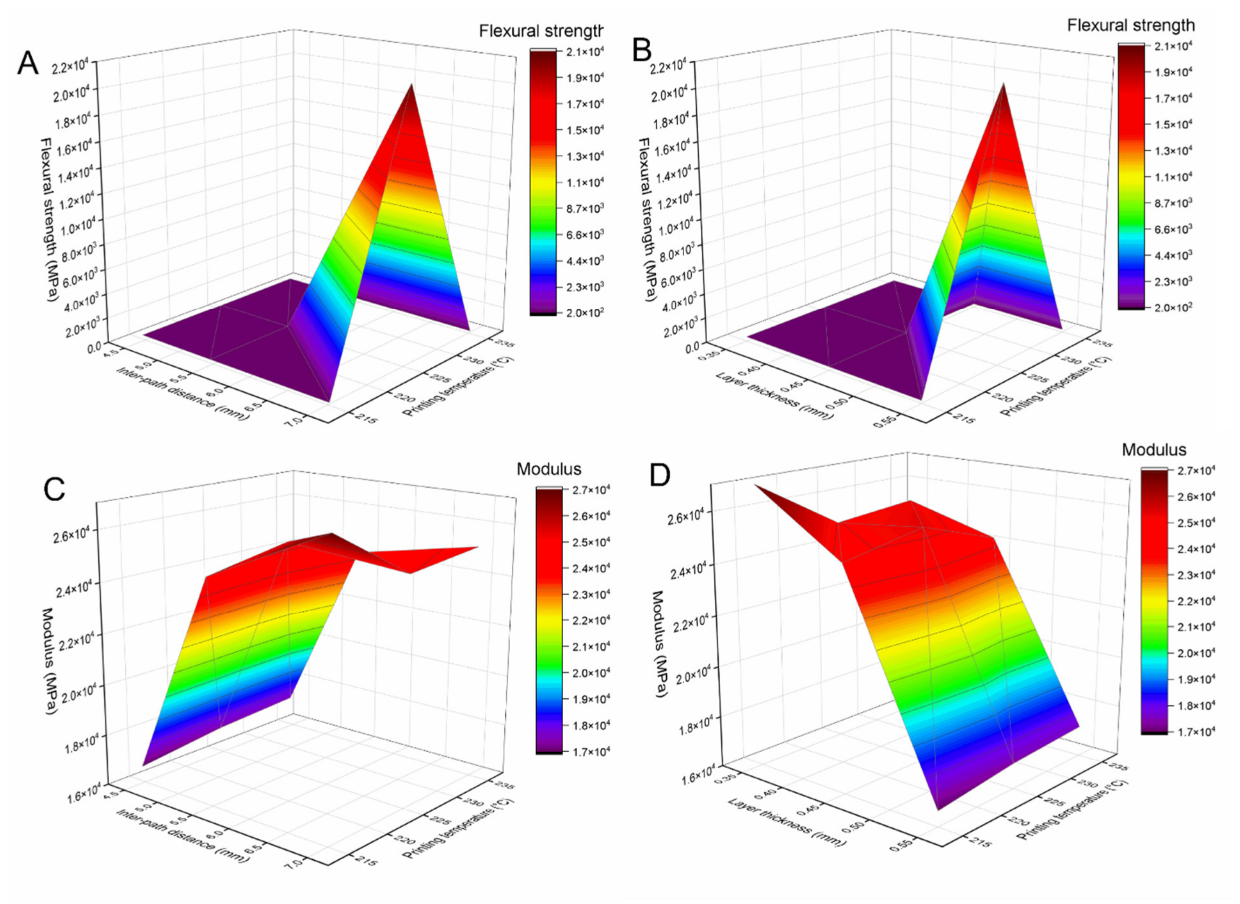

Figure S15. Response surface graphs of the relationship between A: Flexural strength σ_{flex}, inter-path distance, and printing temperature, and B: Flexural strength σ_{flex}, layer thickness, and printing temperature, C: Modulus E_{flex}, inter-path distance, and printing temperature, and D: Modulus E_{flex}, layer thickness, and printing temperature of the samples Specimens from F001 Printing conditions P001-P066 with D=1.1 mm (PP as a polymer matrix).

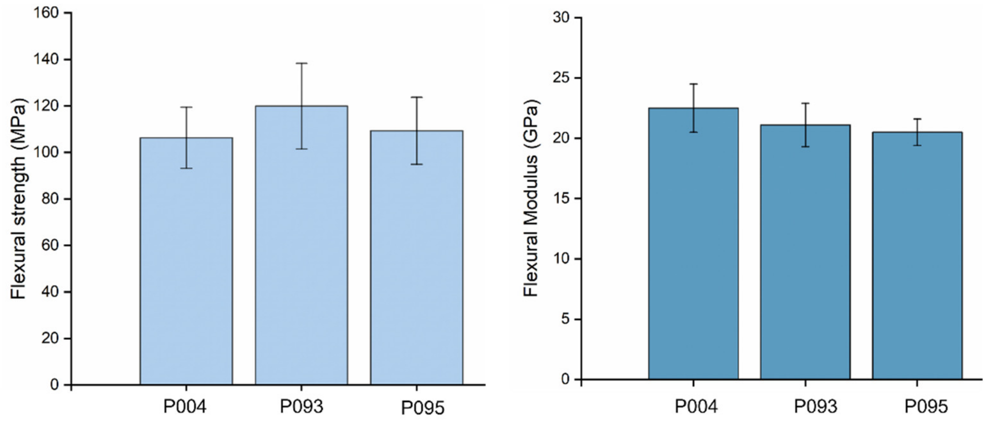

Figure S16. Flexural strength σ_{flex} and Flexural modulus E_{flex} of plasma-treated and untreated cCF-PP (Specimens F001, F002, and F003). The Printing conditions are highlighted in the plots.

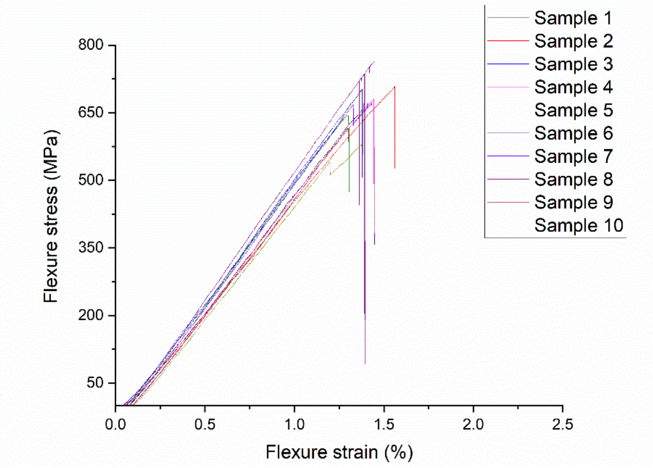

Figure S17. The sample failure mode of F013 Printing conditions P126 with D=0.9 mm (PA12 as a matrix).

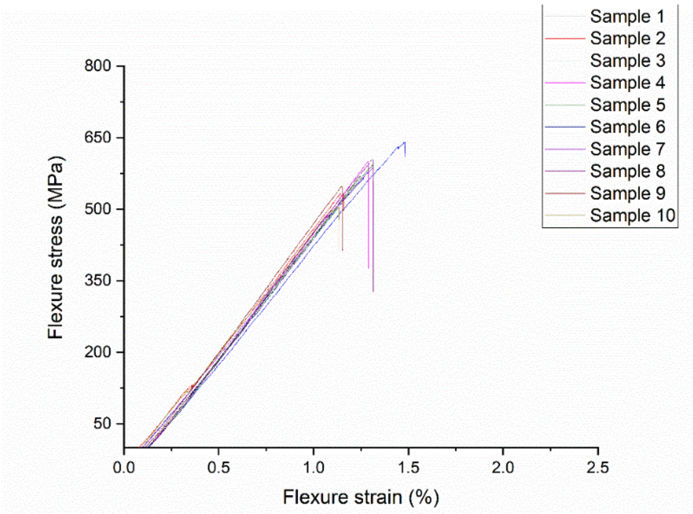

Figure S18. The sample failure mode of F014 Printing conditions P164 with D=0.7 mm (PA12 as a matrix).

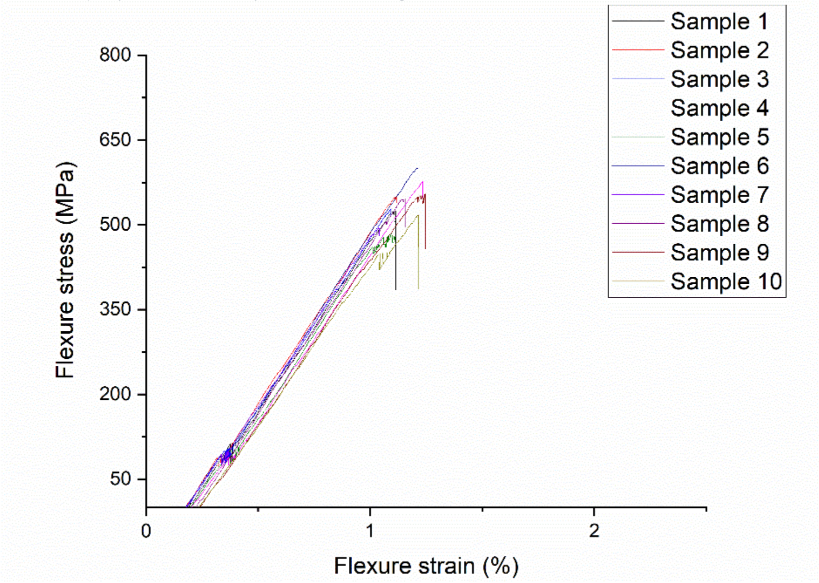

Figure S19. The sample failure mode of F017 Printing conditions P190 with D=0.6 mm (PA12 as a matrix).

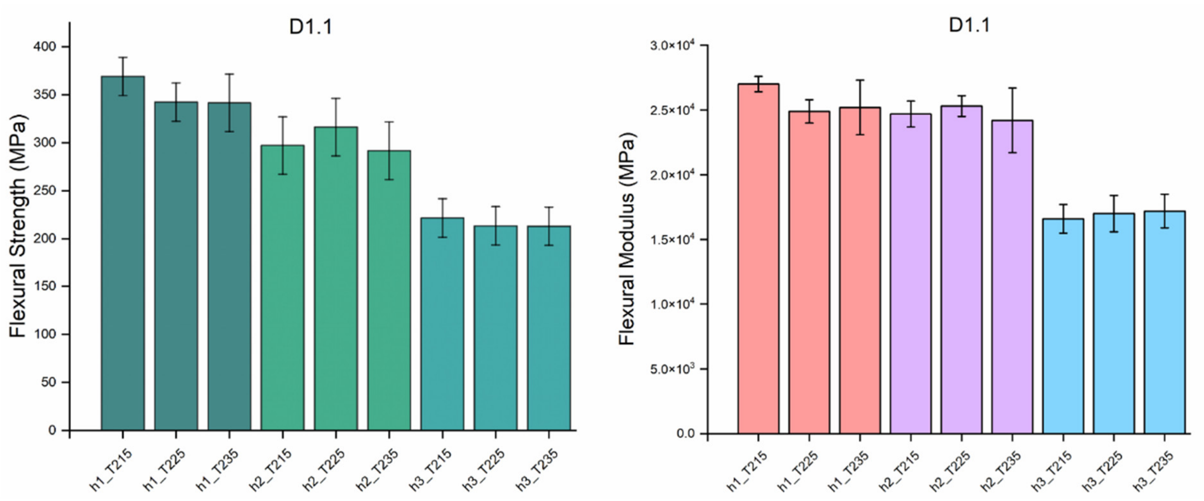

Figure S20. Flexural strength and modulus of the samples with filament diameter D=1.1 mm.

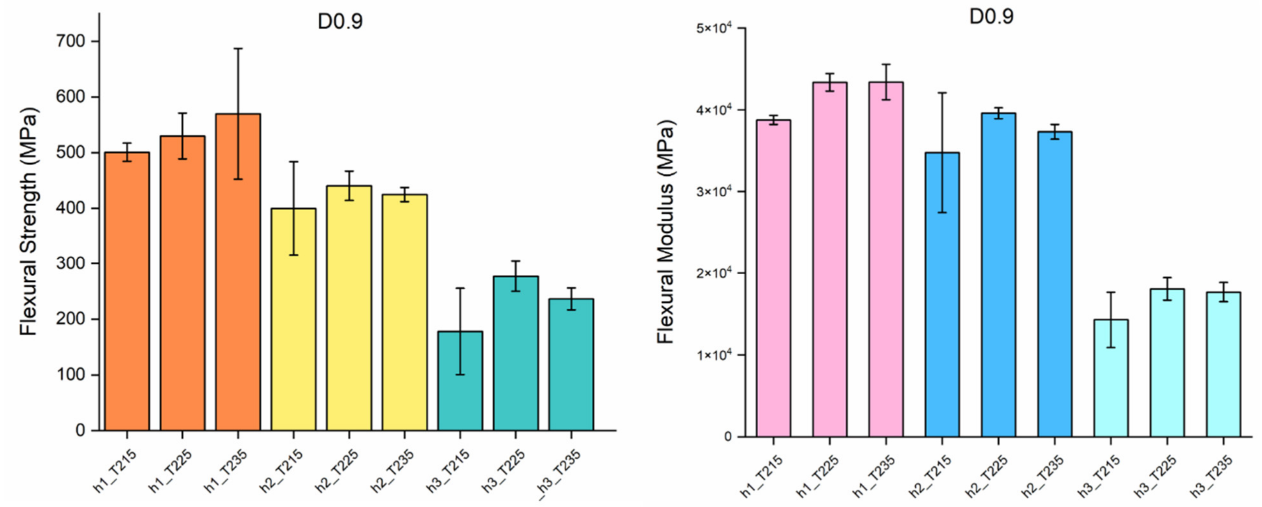

Figure S21. Flexural strength and modulus of the samples with D=0.9 mm.

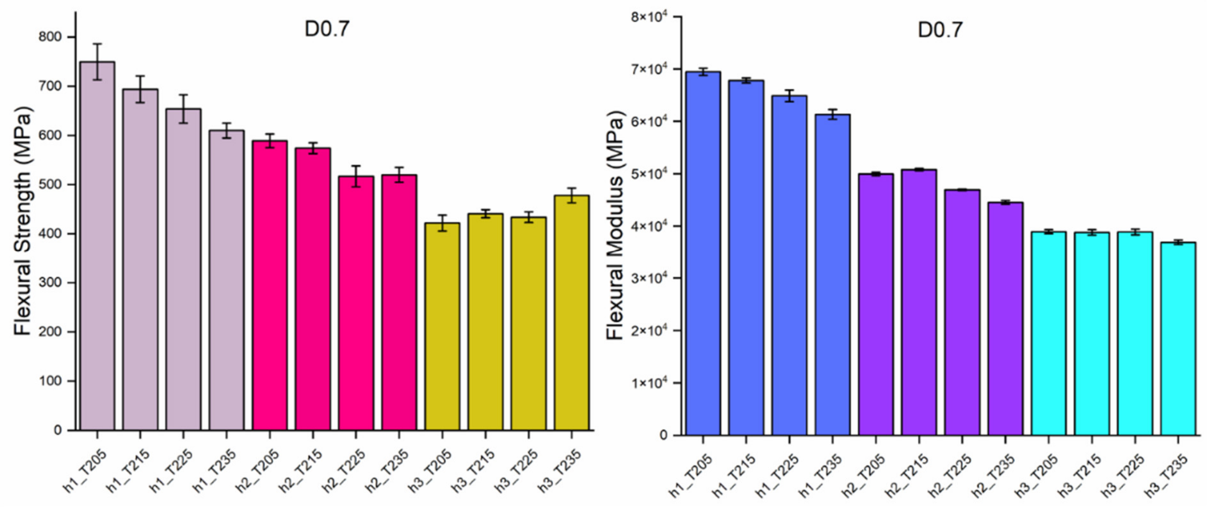

Figure S22. Flexural strength and modulus of the samples with D=0.7 mm.

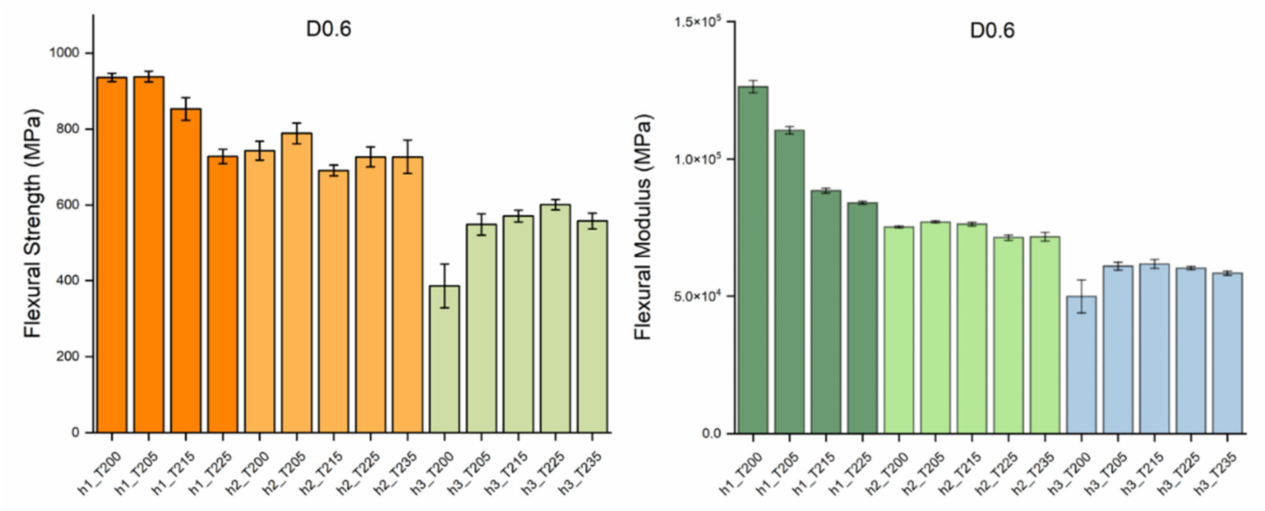

Figure S23. Flexural strength and modulus of the samples with D=0.6 mm.

Table S5. Flexural strength and modulus of the samples with different D, T,s, and h.

D (mm)	Sample Code	Flexural Modulus (GPa)	Flexural Strength (GPa)
**1.1**	D1.1_*h1_T215	27 ± 0.6	0.40 ± 0.02
	D1.1_h1_T225	25 ± 0.9	0.34 ± 0.02
	D1.1_h1_T235	25 ± 2.1	0.34 ± 0.03
	D1.1_*h2_T215	25 ± 1.0	0.30 ± 0.03
	D1.1_h2_T225	25 ± 0.8	0.31 ± 0.03
	D1.1_h2_T235	24 ± 2.5	0.30 ± 0.03
	D1.1_*h3_T215	17 ± 1.1	0.22 ± 0.02
	D1.1_h3_T225	17 ± 1.4	0.21 ± 0.02
	D1.1_h3_T235	17 ± 1.3	0.21 ± 0.02
**0.9**	D0.9_h1_T215	39 ± 0.5	0.5 ± 0.01
	D0.9_h1_T225	43 ± 1.0	0.5 ± 0.04
	D0.9_h1_T235	43 ± 2.1	0.6 ± 0.12
	D0.9_h2_T215	35 ± 7.3	0.4 ± 0.08
	D0.9_h2_T225	40 ± 0.6	0.4 ± 0.03
	D0.9_h2_T235	37 ± 0.9	0.4 ± 0.01
	D0.9_h3_T215	14 ± 3.3	0.2 ± 0.08
	D0.9_h3_T225	18 ± 1.3	0.3 ± 0.03
	D0.9_h3_T235	18 ± 1.1	0.2 ± 0.01
**0.7**	D0.7_h1_T205	69 ± 0.7	0.7 ± 0.04
	D0.7_h1_T215	68 ± 0.5	0.7 ± 0.03
	D0.7_h1_T225	65 ± 1.1	0.7 ± 0.03
	D0.7_h1_T235	61 ± 0.9	0.6 ± 0.02
	D0.7_h2_T205	50 ± 0.3	0.6 ± 0.01
	D0.7_h2_T215	51 ± 0.2	0.6 ± 0.01

	D0.7_h2_T225	47 ± 0.1	0.5 ± 0.02
	D0.7_h2_T235	45 ± 0.4	0.5 ± 0.02
	D0.7_h3_T205	39 ± 0.4	0.4 ± 0.02
	D0.7_h3_T215	39 ± 0.5	0.4 ± 0.01
	D0.7_h3_T225	39 ± 0.5	0.4 ± 0.01
	D0.7_h3_T235	37 ± 0.4	0.5 ± 0.02
**0.6**	D0.6_h1_T200	126 ± 2.2	0.9 ± 0.11
	D0.6_h1_T205	110 ± 1.3	0.9 ± 0.01
	D0.6_h1_T215	88 ± 0.9	0.9 ± 0.03
	D0.6_h1_T225	84 ± 0.5	0.7 ± 0.02
	D0.6_h2_T200	75 ± 0.4	0.7 ± 0.03
	D0.6_h2_T205	77 ± 0.4	0.8 ± 0.03
	D0.6_h2_T215	76 ± 0.7	0.7 ± 0.01
	D0.6_h2_T225	71 ± 0.9	0.7 ± 0.03
	D0.6_h2_T235	71 ± 1.5	0.7 ± 0.04
	D0.6_h3_T200	50 ± 1.9	0.4 ± 0.06
	D0.6_h3_T205	61 ± 1.4	0.5 ± 0.03
	D0.6_h3_T215	62 ± 1.6	0.6 ± 0.02
	D0.6_h3_T225	60 ± 0.6	0.6 ± 0.01
	D0.6_h3_T235	58 ± 0.7	0.6 ± 0.02

* **h1** *means h1 & s1,* **h2** *means h2 & s2,* **h3** *means h3 & s3*

DSC analysis for the determination of the annealing parameters.

Table S6. DSC tests parameters.

	End Temperature (°C)	Heating/cooling rate (K/min)	Isotherm (min)
**Start**	25	10	
**Heating**	250	10	10
**Cooling**	25	10	10
**Heating**	250	10	10
**Cooling**	25	10	

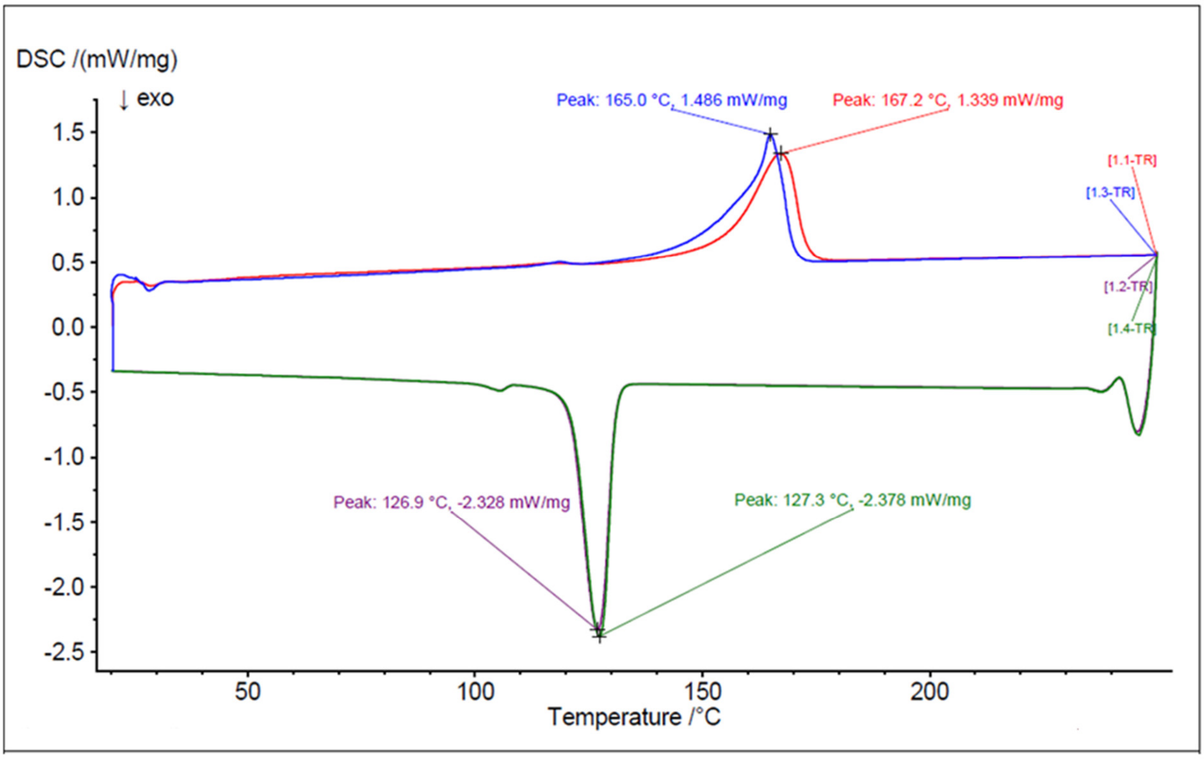

Figure S24. DSC results of PP.

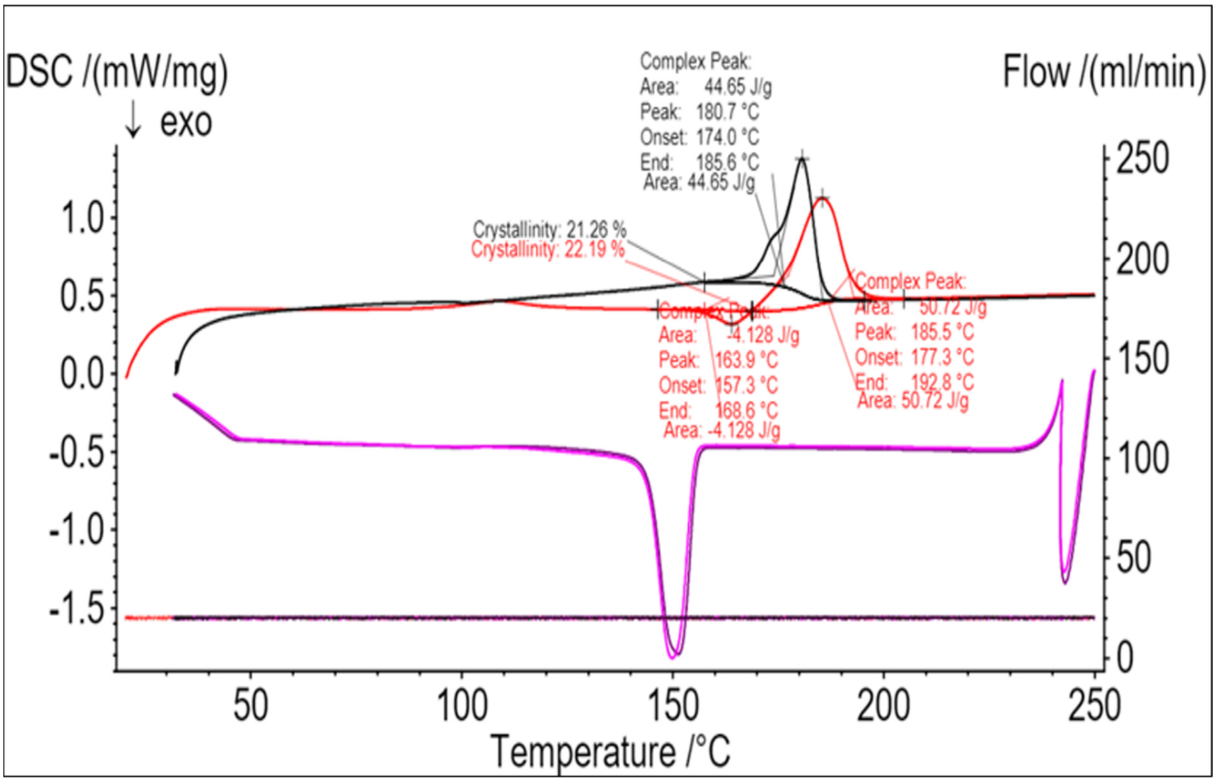

Figure S25. DSC results of PA12.

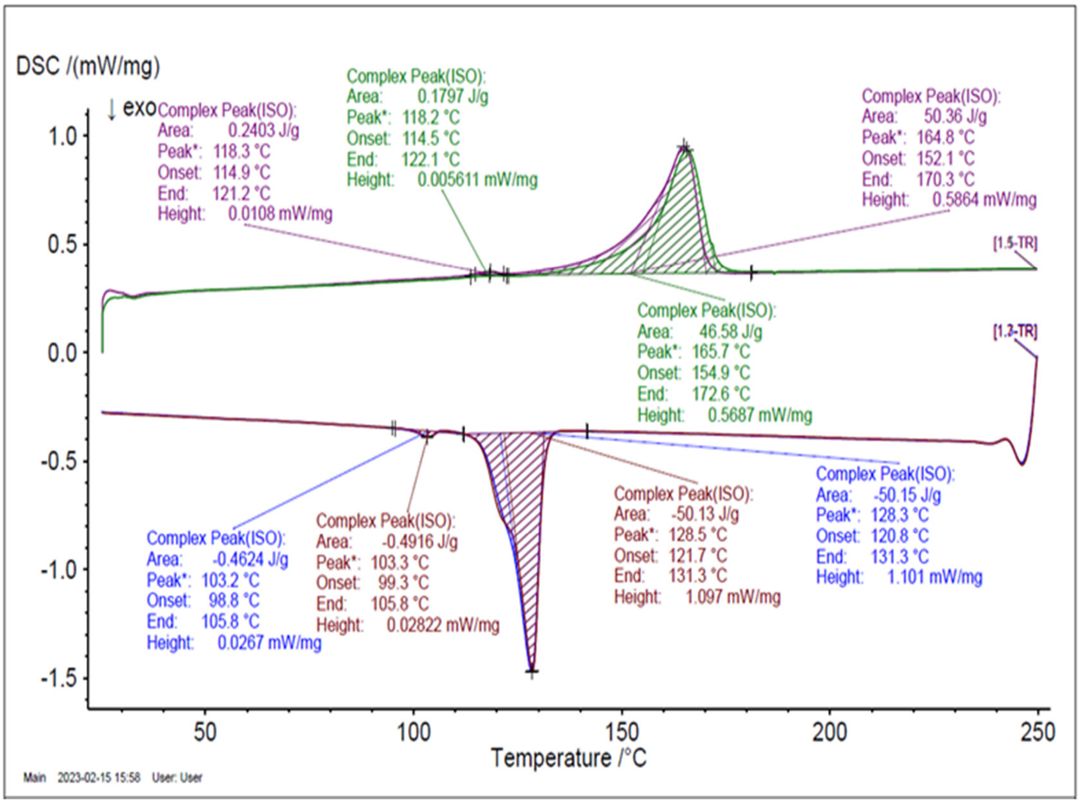

*Figure S26. DSC of unannealed cCF-PP (peak *:the location of the peak maximum).*

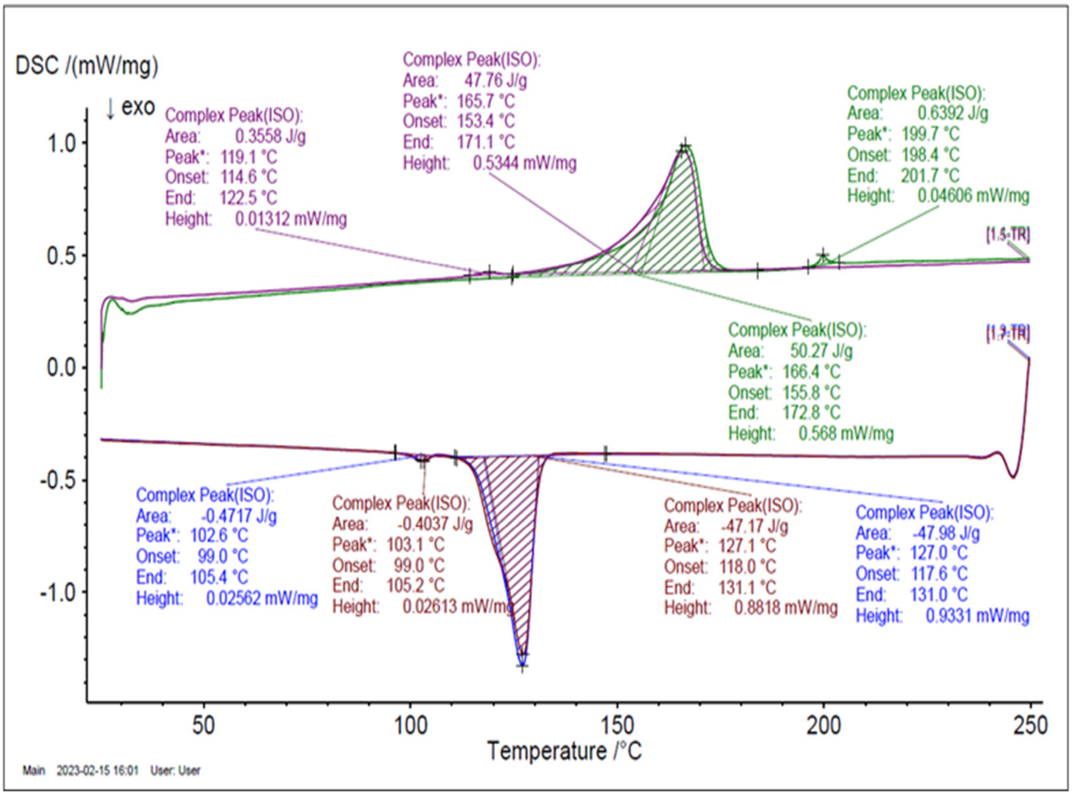

*Figure S27. DSC of annealed cCF-PP (peak *:the location of the peak maximum).*

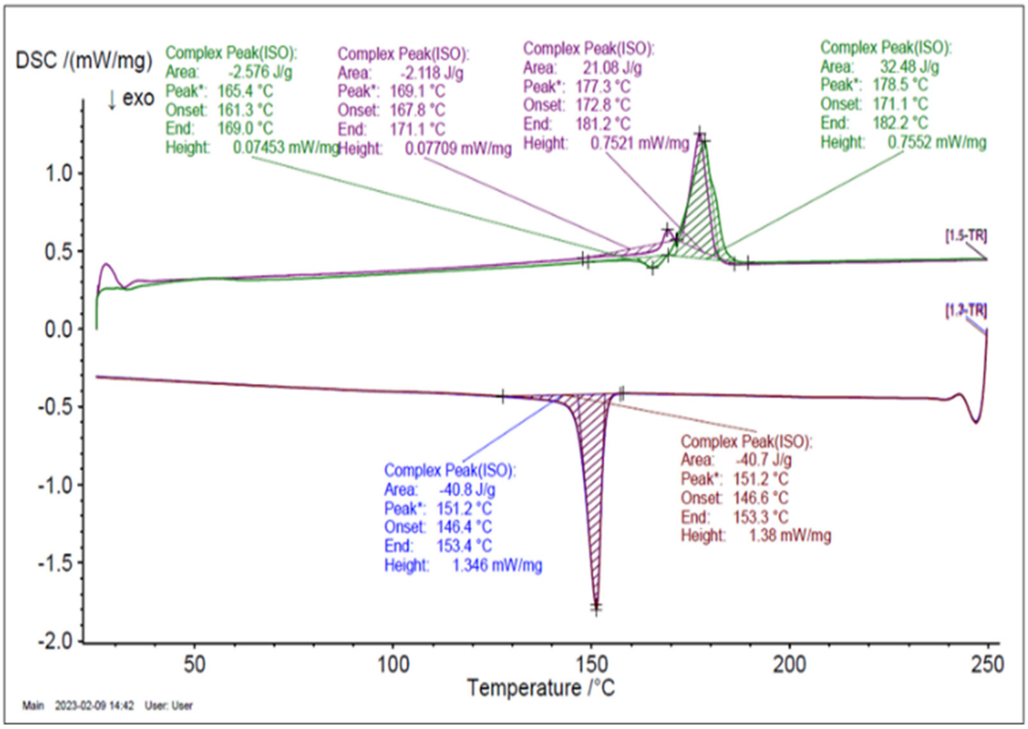

*Figure S28. DSC result of unannealed cCF-PA12 (peak *:the location of the peak maximum).*

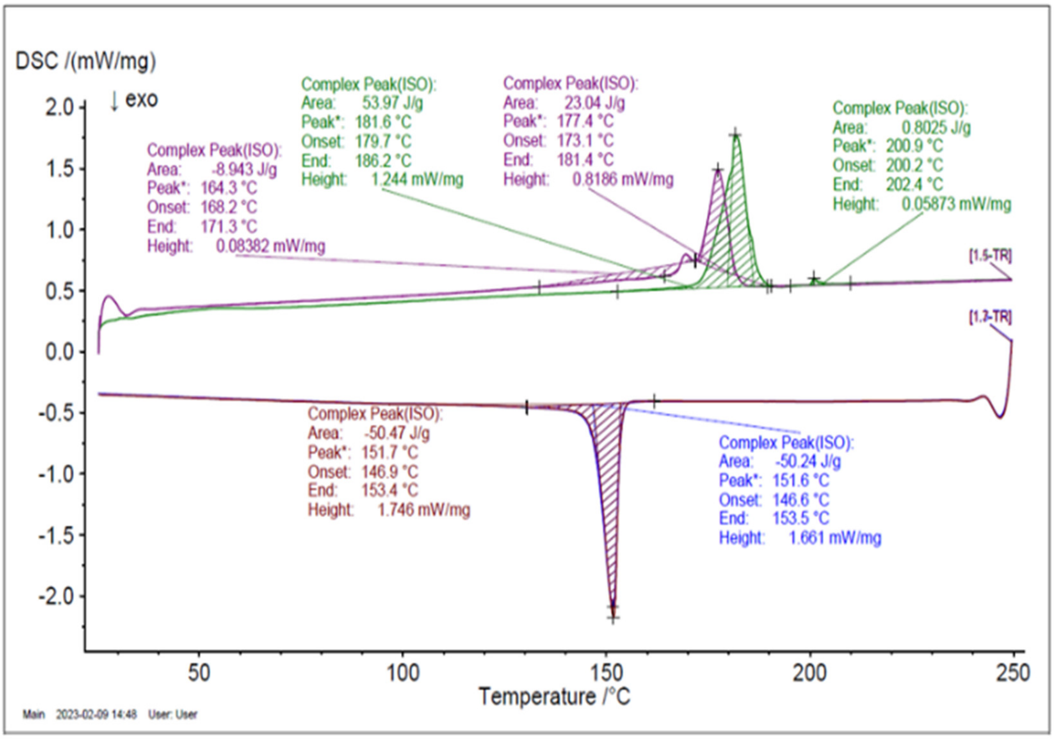

*Figure S29. DSC result of annealed cCF-PA12 (peak *:the location of the peak maximum).*

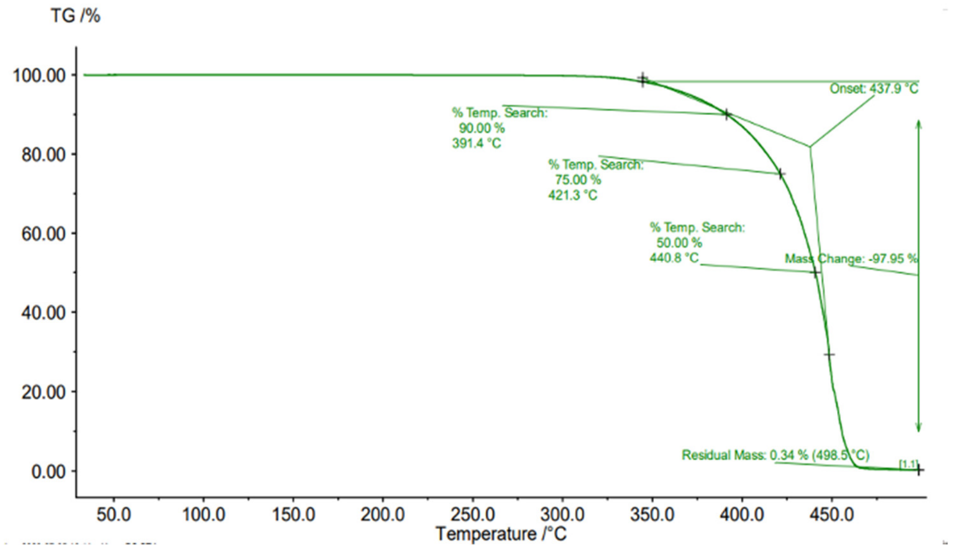

Figure S30. TGA result of PP.

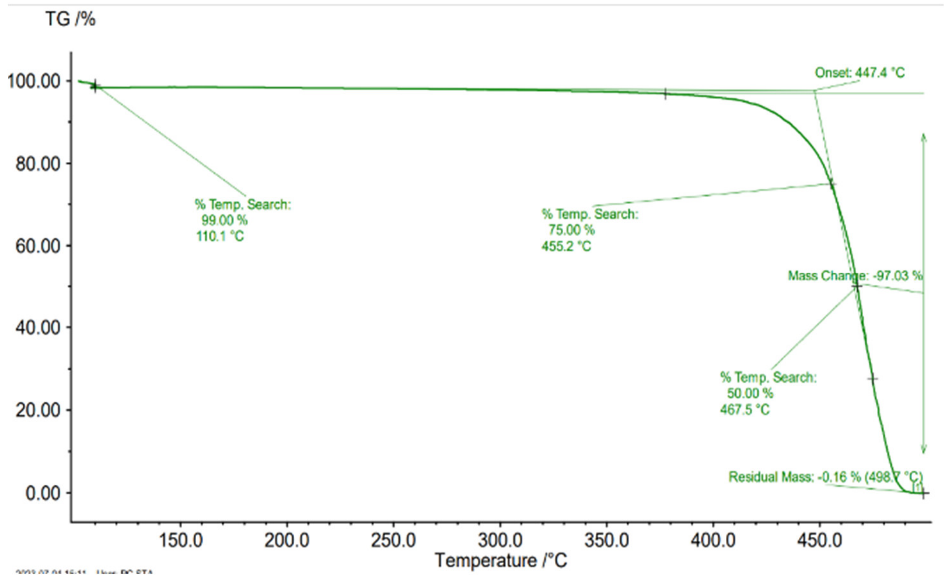

Figure S31. TGA result of PA12.

Table S7. Estimated flexural properties of the cCF composites for varying fiber fractions.

v_f (%)	cCF-PA12		cCF-PP	
	E_{flex}(GPa)	σ_{flex}(GPa)	E_{flex}(GPa)	σ_{flex}(GPa)
**10**	21.70	0.3	14.10	0.08
**20**	42.10	0.6	27.60	0.16
**30**	62.40	0.8	41.10	0.24
**40**	82.80	1.1	54.60	0.32
**50**	103.20	1.3	68.10	0.40

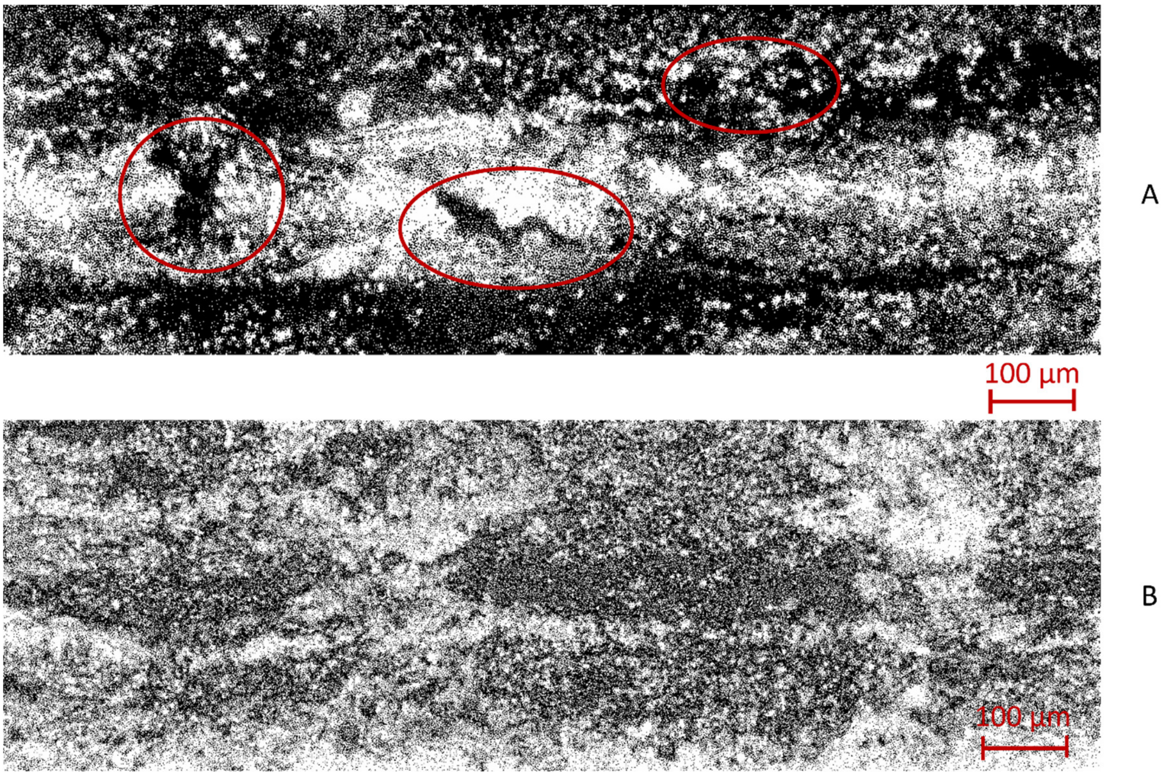

Figure S32. ImageJ results of A. Non-annealed cCF-PP (P62) sample and B. An annealed cCF-PP sample at 140 °C.

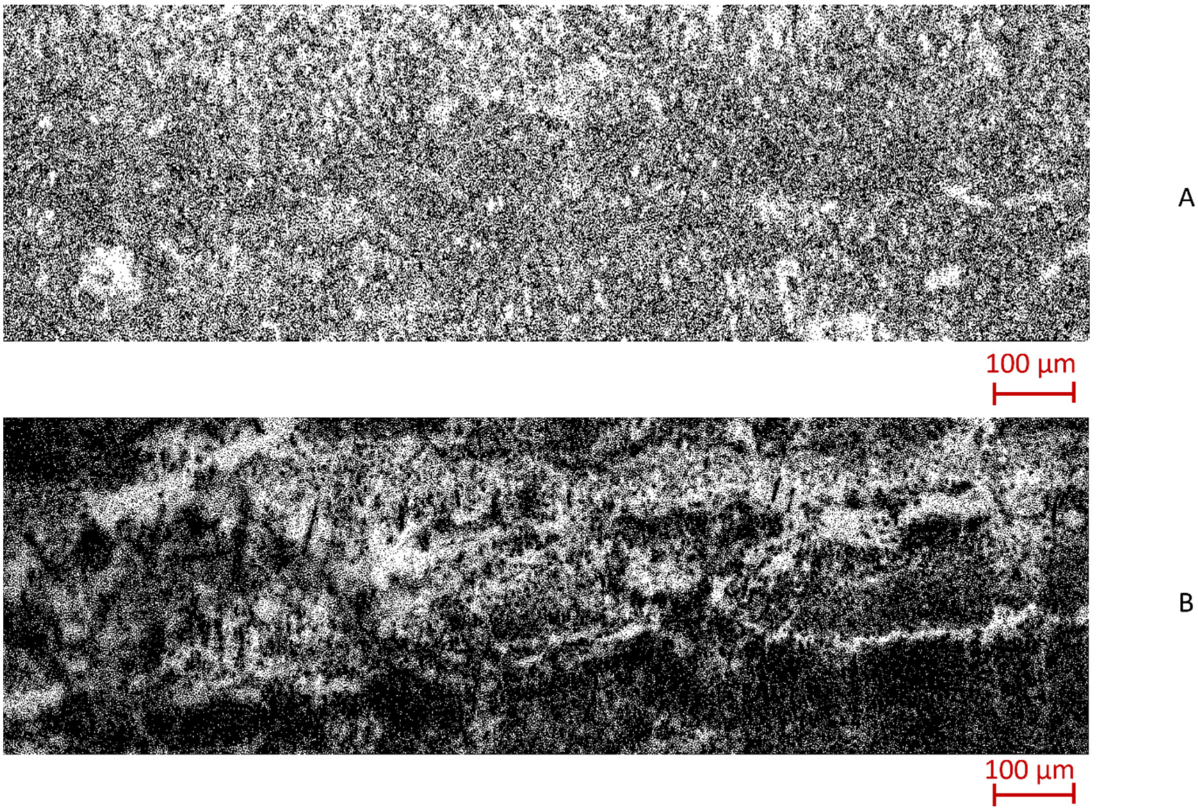

Figure S33. ImageJ results of A. Non-annealed cCF-PA12 (P142) sample and B. An annealed cCF-PA12 sample at 165 °C.

Table S8. Printing conditions for the test specimens.

Name	T_nozzle [°C]	T_bed [°C]	*s* [mm]	*h* [mm]	Filament	*v*_line [mm/min]	*v*_curve [mm/min]
P001	230	60	1.30	0.45	F001	60	60
P002	230	80	1.67	0.35	F001	120	30
P003	230	80	1.30	0.45	F001	60	60
P004	230	80	1.30	0.45	F001	120	30
P005	230	80	1.06	0.55	F001	120	30
P006	230	80	1.06	0.55	F001	60	60
P007	230	80	1.06	0.55	F001	60	60
P008	230	80	1.06	0.55	F001	60	60
P009	230	80	1.06	0.55	F001	60	60
P010	230	80	1.06	0.55	F001	60	60
P011	230	60	1.30	0.45	F001	60	60
P012	240	80	1.06	0.55	F001	60	60
P013	235	80	1.06	0.55	F001	60	60
P014	235	80	1.06	0.55	F001	60	60
P015	240	80	1.06	0.55	F001	60	60
P016	245	80	1.06	0.55	F001	60	60
P017	245	80	1.06	0.55	F001	60	60
P018	220	80	1.06	0.55	F001	60	60
P019	210	80	1.06	0.55	F001	60	60
P020	210	80	1.06	0.55	F001	60	60
P021	200	80	1.06	0.55	F001	60	60
P022	200	80	1.06	0.55	F001	60	60
P023	220	80	1.06	0.55	F001	60	60
P024	220	80	1.06	0.55	F001	60	60
P025	220	80	1.06	0.55	F001	120	60
P026	230	80	1.06	0.55	F001	120	60
P027	230	80	1.06	0.55	F001	120	60
P028	230	80	1.06	0.55	F001	120	60
P029	230	80	1.06	0.55	F001	120	60
P030	230	80	1.06	0.55	F001	120	60
P031	230	80	1.06	0.55	F001	120	30
P032	230	80	1.06	0.55	F001	120	30
P033	230	80	1.06	0.55	F001	120	30
P034	230	80	1.06	0.55	F001	120	30
P035	230	80	1.06	0.55	F001	120	30
P036	230	80	1.06	0.55	F001	120	30
P037	230	80	1.30	0.45	F001	120	30
P038	230	80	1.30	0.45	F001	120	30
P039	230	80	1.30	0.45	F001	120	30
P040	230	80	1.30	0.45	F001	120	30
P041	230	80	1.30	0.45	F001	120	30
P042	230	80	1.30	0.45	F001	120	30

P043	230	80	1.30	0.45	F001	120	30
P044	230	80	1.30	0.45	F001	120	30
P045	230	80	1.30	0.45	F001	120	30
P046	235	80	1.30	0.45	F001	120	30
P047	235	80	1.30	0.45	F001	120	30
P048	235	80	1.30	0.45	F001	120	30
P049	235	80	1.30	0.45	F001	120	30
P050	235	80	1.30	0.45	F001	120	30
P051	235	80	1.30	0.45	F001	120	30
P052	235	85	1.30	0.45	F001	120	30
P053	235	85	1.30	0.45	F001	120	30
P054	235	85	1.30	0.45	F001	120	30
P055	235	85	1.30	0.45	F001	120	30
P056	235	85	1.30	0.45	F001	120	30
P057	235	85	1.30	0.45	F001	120	30
P058	235	85	1.30	0.45	F001	120	30
P059	235	85	1.30	0.45	F001	120	30
P060	235	85	1.30	0.45	F001	120	30
P061	235	85	1.30	0.45	F001	120	30
P062	235	85	1.30	0.45	F001	120	30
p063	235	85	1.30	0.45	F001	120	30
P064	235	85	1.30	0.45	F001	120	30
P065	235	85	1.30	0.45	F001	120	30
P066	235	85	1.30	0.45	F001	120	30
P067	235	110	1.30	0.45	F005	120	30
P068	235	110	1.30	0.45	F005	120	30
P069	235	110	1.30	0.45	F005	120	30
P070	235	110	1.30	0.45	F005	120	30
P071	235	110	1.30	0.45	F005	120	30
P072	235	110	1.30	0.45	F005	120	30
P073	245	110	1.30	0.45	F005	120	30
P074	245	110	1.30	0.45	F005	120	30
P075	225	110	1.30	0.45	F005	120	30
P076	225	110	1.30	0.45	F005	120	30
P077	245	110	1.30	0.45	F005	120	30
P078	245	110	1.30	0.45	F005	120	30
P079	245	110	1.30	0.45	F005	120	30
P080	235	110	1.30	0.45	F005	120	30
P081	235	110	1.30	0.45	F005	120	30
P082	235	110	1.30	0.45	F005	120	30
P083	245	110	1.60	0.55	F005	120	30
P084	245	110	1.40	0.55	F005	120	30
P085	245	110	1.40	0.55	F005	120	30
P086	235	80	1.40	0.45	F004	120	30
P087	235	80	1.40	0.45	F004	120	30
P088	235	80	1.30	0.45	F004	120	30
P089	235	80	1.30	0.45	F004	120	30

P090	235	80	1.40	0.45	F004	120	30
P091	235	80	1.40	0.45	F004	120	30
P092	235	80	1.30	0.45	F004	120	30
P093	235	80	1.30	0.45	F002	120	30
P094	235	80	1.30	0.45	F003	120	30
P095	235	80	1.30	0.45	F003	120	30
P096	235	80	1.30	0.45	F002	120	30
P097	235	80	1.30	0.45	F004	120	30
P098	235	80	1.30	0.45	F004	120	30
P099	235	80	1.30	0.45	F004	120	30
P100	235	80	1.30	0.45	F004	120	30
P101	235	80	1.30	0.45	F004	120	30
P102	235	80	1.30	0.45	F004	120	30
P103	235	110	1.30	0.45	F008	120	30
P104	235	110	1.30	0.45	F008	120	30
P105	235	110	1.30	0.45	F006	120	30
P106	245	110	1.30	0.45	F006	120	30
P107	225	110	1.30	0.45	F007	120	30
P108	225	110	1.30	0.45	F007	120	30
P109	225	110	1.30	0.45	F007	120	30
P110	215	110	1.30	0.45	F007	120	30
P111	215	110	1.30	0.45	F007	120	30
P112	215	110	1.30	0.45	F007	120	30
P113	215	110	1.67	0.35	F009	120	30
P114	215	110	1.67	0.35	F009	120	30
P115	215	110	1.67	0.35	F009	120	30
P116	215	110	1.67	0.35	F011	120	30
P117	215	110	1.67	0.35	F011	120	30
P118	215	110	1.67	0.35	F011	120	30
P119	215	110	1.67	0.35	F011	120	30
P120	215	110	1.67	0.35	F012	120	30
P121	215	110	1.67	0.35	F012	120	30
P122	215	110	1.67	0.35	F010	120	30
P123	215	110	1.67	0.35	F010	120	30
P124	215	110	1.67	0.35	F010	120	30
P125	215	110	1.67	0.35	F010	120	30
P126	215	110	1.37	0.29	F013	120	30
P127	215	110	1.37	0.29	F013	120	30
P128	215	110	1.37	0.29	F013	120	30
P129	225	110	1.37	0.29	F013	120	30
P130	225	110	1.37	0.29	F013	120	30
P131	225	110	1.37	0.29	F013	120	30
P132	235	110	1.37	0.29	F013	120	30
P133	235	110	1.37	0.29	F013	120	30
P134	235	110	1.37	0.29	F013	120	30
P135	235	110	1.06	0.37	F013	120	30
P136	235	110	1.06	0.37	F013	120	30

P137	235	110	1.06	0.37	F013	120	30
P138	235	110	1.06	0.37	F013	120	30
P139	225	110	1.06	0.37	F013	120	30
P140	225	110	1.06	0.37	F013	120	30
P141	225	110	1.06	0.37	F013	120	30
P142	215	110	1.06	0.37	F013	120	30
P143	215	110	1.06	0.37	F013	120	30
P144	215	110	1.06	0.37	F013	120	30
P145	215	110	0.87	0.45	F013	120	30
P146	215	110	0.87	0.45	F013	120	30
P147	215	110	0.87	0.45	F013	120	30
P148	225	110	0.87	0.45	F013	120	30
P149	225	110	0.87	0.45	F013	120	30
P150	225	110	0.87	0.45	F013	120	30
P151	235	110	0.87	0.45	F013	120	30
P152	235	110	0.87	0.45	F013	120	30
P153	235	110	0.87	0.45	F013	120	30
P154	215	110	1.06	0.22	F014	120	30
P155	215	110	1.06	0.22	F014	120	30
P156	215	110	1.06	0.22	F014	120	30
P157	225	110	1.06	0.22	F014	120	30
P158	225	110	1.06	0.22	F014	120	30
P159	235	110	1.06	0.22	F014	120	30
P160	235	110	1.06	0.22	F014	120	30
P161	205	110	1.06	0.22	F014	120	30
P162	205	110	1.06	0.22	F014	120	30
P163	205	110	1.06	0.22	F014	120	30
P164	215	110	0.83	0.29	F014	120	30
P165	215	110	0.83	0.29	F014	120	30
P166	225	110	0.83	0.29	F014	120	30
P167	225	110	0.83	0.29	F014	120	30
P168	235	110	0.83	0.29	F014	120	30
P169	235	110	0.83	0.29	F014	120	30
P170	205	110	0.83	0.29	F014	120	30
P171	205	110	0.83	0.29	F014	120	30
P172	205	110	0.68	0.35	F014	120	30
P173	205	110	0.68	0.35	F014	120	30
P174	215	110	0.68	0.35	F014	120	30
P175	215	110	0.68	0.35	F014	120	30
P176	225	110	0.68	0.35	F014	120	30
P177	225	110	0.68	0.35	F014	120	30
P178	235	110	0.68	0.35	F014	120	30
P179	235	110	0.68	0.35	F014	120	30
P180	235	110	0.68	0.35	F014	120	30
P181	225	110	0.68	0.35	F014	120	30
P182	215	110	0.68	0.35	F014	120	30
P183	205	110	0.68	0.35	F014	120	30

P184	215	110	0.91	0.19	F016	120	30
P185	215	110	0.71	0.25	F017	120	30
P186	215	110	0.71	0.25	F017	120	30
P187	225	110	0.71	0.25	F017	120	30
P188	225	110	0.71	0.25	F017	120	30
P189	205	110	0.71	0.25	F017	120	30
P190	205	110	0.71	0.25	F017	120	30
P191	195	110	0.71	0.25	F017	120	30
P192	200	110	0.71	0.25	F017	120	30
P193	235	110	0.71	0.25	F017	120	30
P194	235	110	0.71	0.25	F017	120	30
P195	205	110	0.58	0.30	F017	120	30
P196	205	110	0.58	0.30	F017	120	30
P197	205	110	0.58	0.30	F017	120	30
P198	215	110	0.58	0.30	F017	120	30
P199	215	110	0.58	0.30	F017	120	30
P200	225	110	0.58	0.30	F017	120	30
P201	225	110	0.58	0.30	F017	120	30
P202	235	110	0.58	0.30	F017	120	30
P203	235	110	0.58	0.30	F017	120	30
P204	205	110	0.91	0.19	F017	120	30
P205	205	110	0.91	0.19	F017	120	30
P206	215	110	0.91	0.19	F017	120	30
P207	225	110	0.91	0.19	F017	120	30
P208	225	110	0.91	0.19	F017	120	30
P209	235	110	0.91	0.19	F017	120	30
P210	235	110	0.91	0.19	F017	120	30
P211	200	110	0.91	0.19	F017	120	30
P212	200	110	0.91	0.19	F017	120	30
P213	200	110	0.71	0.25	F017	120	30
P214	200	110	0.58	0.3	F017	120	30
P215	200	110	0.58	0.3	F017	120	30
